# Unveiling of climate change-driven decline of suitable habitat for Himalayan bumblebees

**DOI:** 10.1038/s41598-024-52340-9

**Published:** 2024-02-29

**Authors:** Amar Paul Singh, Kritish De, Virendra Prasad Uniyal, Sambandam Sathyakumar

**Affiliations:** 1 Wildlife Institute of India, Chandrabani, Dehradun, Uttarakhand 248001 India; 2 Department of Life Sciences, Sri Sathya Sai University for Human Excellence, Navanihal, Okali Post, Kamalapur, Kalaburagi, Karnataka 585313 India; 3 Graphic Era (Deemed to be) University, Bell Road, Clement Town, Dehradun, Uttarakhand, 248002, India

**Keywords:** Ecology, Biodiversity, Climate-change ecology, Conservation biology, Ecological modelling

## Abstract

Insect pollinators, especially bumblebees are rapidly declining from their natural habitat in the mountain and temperate regions of the world due to climate change and other anthropogenic activities. We still lack reliable information about the current and future habitat conditions of bumblebees in the Himalaya. In this study, we used the maximum entropy algorithm for SDM to look at current and future (in 2050 and 2070) suitable habitats for bumblebees in the Himalaya. We found that the habitat conditions in the Himalayan mountain range do not have a very promising future as suitable habitat for most species will decrease over the next 50 years. By 2050, less than 10% of the Himalayan area will remain a suitable habitat for about 72% of species, and by 2070 this number will be raised to 75%. During this time period, the existing suitable habitat of bumblebees will be declined but some species will find new suitable habitat which clearly indicates possibility of habitat range shift by Himalayan bumblebees. Overall, about 15% of the Himalayan region is currently highly suitable for bumblebees, which should be considered as priority areas for the conservation of these pollinators. Since suitable habitats for bumblebees lie between several countries, nations that share international borders in the Himalayan region should have international agreements for comprehensive pollinator diversity conservation to protect these indispensable ecosystem service providers.

## Introduction

Bumblebees (Hymenoptera: Apidae: Bombus Latreille) are cold-adapted insect pollinators with thermoregulatory abilities that allow them to be active in low ambient temperatures^1^. Especially at high altitudes, where the pollination power of other pollinators becomes limited by extreme environmental conditions such as low temperatures and little oxygen, bumblebees play an important pollination role due to their extreme altitude adaptations^2^. Because of this ability, they are recognised as the most important pollinators of wild and agricultural plant species in cold habitats such as mountain systems and temperate zones^1,3–5^. Most bumblebees have an annual life cycle that generally begins after a hibernation period when fertilized queens look for a place to nest^6^. These queens begin by laying eggs that progress through several larval instars to pupate into workers that perform labour tasks for the colony, including foraging for pollen and nectar from a wide variety of flowering plants^7^. Male and fertile female bees are produced later in the season, usually in summer. This annual cycle ends with the start of winter, when the newly produced and fertilized queens seek underground shelter to survive the winter while the other remaining bees die^8^. Although bumblebees feed on a variety of flowers, their food choices vary depending on morphology, availability of floral resources, and competitors. For example, long-tongued bumblebees prefer to feed on flowers with deep corolla tubes, while short-tongued species have broader dietary preferences^9^. To date, 265 species of bumblebee (genus: *Bombus*) have been discovered worldwide, including 53 species from the Himalayas, of which nine species are endemic to this region (https://bumblebeespecialistgroup.org).

Because insects are the most diverse group of animals on the planet, with more than a million described species accounting for more than half of all known multicellular organisms, how insects will respond to global climate change due to increased temperatures, increased atmospheric CO_2_ levels and changes in precipitation patterns is of particular interest to scientists^10^. Recent studies have revealed an alarming decline in insect species, particularly flying insect species, in the northern temperate region for the reasons mentioned above, all of which will have a significant impact on the ecosystem services provided by insects^11^. Such impacts will be magnified many times over by climate change-related impacts on insect species, including phenological changes (e.g., earlier flight times, improved winter survival, and changes in developmental rate), range shifts to regions outside their current thermal tolerances, and competition with invasive species11. Due to their habitat and behavioural specialization, bumblebee populations in the temperate and mountainous regions of Europe and North America are negatively affected by climate change, urbanization, increasing agricultural activity, pesticide use, changes in land use, pathogen pressure and exotic species, resulting in rapid population declines^12–17^. This population decline may result in a loss of acute pollination services for flowering plants and potentially promote an extinction vortex between pollinators and the plants pollinated by that particular species^18,19^.

The mountains are among the most ecologically functional areas and provide a wide range of ecosystem services (ES) to the surrounding population, but these are extremely vulnerable due to climate change and land use and land cover modifications^20^. The Himalayan Mountains host a complex topographical and climatic diversity and are home to one of the richest plant species in the world, the species distribution pattern of which depends on the distribution pattern of wild pollinators^21^. The Himalaya experienced significant warming between 1951 and 2014 (0.2 °C/decade), particularly at higher elevations (0.5 °C/decade) and climate projections suggest that the average temperature will rise by 2–5 °C by 2050, with annual rainfall increasing up to 12% by 2050^22^. Climate change may irreversibly affect ecosystems and biodiversity, leading to the extinction of several Himalayan species, particularly of pollinating insects by altering their behaviour, physiology and phenological coevality with host plants. Although it was predicted that climate change would reduce potentially suitable habitat for bumblebees in the Great Himalayan National Park Conservation Area^23^, no attempt has yet been made to integrate different climate change scenarios to quantify future changes in habitats of individual bumblebee species across the Himalaya. In this work, we quantified changes in bumblebee habitat suitability due to climate change impacts at the individual species level in the Himalaya. Besides, by overlaying the currently suitable habitats of different species of bumblebees, we prepared a model showing the presence of ‘hotspots’ or ‘conservation priority areas’ in the Himalaya where conservation management activities can simultaneously benefit many species.

## Methods

We performed this study in the Himalaya and the Trans-Himalayan region of India. The entire study region extends from 37.088362° N to 26.395343° S and 72.513077° W to 97.412895° E. This physically and biologically complex and diversified mountain system is characterized by high biodiversity, undulating physical settings, and varied climatic regimes^24^. The Himalayan mountain region is comprised of four biogeographic provinces namely North-West Himalaya, West Himalaya, Central Himalaya and Eastern Himalaya^25^. The Trans-Himalayan region is the ‘High-Altitude Cold Desert Zone’ which is further classified into three zones namely Ladakh mountains (which includes Kargil, Nubra and Zanskar in the union territory of Ladakh and Lahaul-Spiti and Kinnaur in Himachal Pradesh), Tibetan plateau (which includes Changthang region of union territory of Ladakh and northern part of Uttarakhand) and Sikkim plateau^26^.

We conducted field work in Western Himalaya (at Great Himalayan National Park Conservation Area) and Trans-Himalayan region (at Lahaul and Ladakh area) from May, 2018 to February, 2020. We visited each site once during this period. At Great Himalayan National Park Conservation Area we collected samples from Parvati Valley (31.926128° N, 77.472569° S), Tirthan Valley (31.601248° N, 77.480223° E), Sainj Valley 31.769419° N, 77.288721° E and Jiwa Nala Valley (31.874146° N, 77.444971° E). At Ladakh region we collected samples from Shyok Valley (34.695838° N, 77.265009° E), Nubra Valley (34.769386° N, 77.532645° S) and Indus Valley (34.189607° N, 77.330468° S). At Lahul region we collected samples from Bhaga Valley (32.648904° N, 77.199401° E), Chandra Valley (32.406882° N, 77.249990° E), Miyar Valley (32.825168° N, 76.743680° E) and Chenab Valley (32.625528° N, 76.8726743° S).

The Great Himalayan National Park extends from the Himalayan foothills to the Alpine zone ranging from 1300 to 6000 m of elevational gradient and is characterised by temperate broad leaf forests, pine forests and arid alpine meadows and pastures at higher elevational zones with relatively low annual rainfall^23^. The Trans-Himalayan region, ranging from 3000 to 7000 m of elevational gradient, is characterised by very harsh and extreme environmental conditions characterised by scanty rainfall, massive snowfall, early frost damage, high wind velocity, reduced oxygen concentration, a short growing season, and low and fluctuating temperature ranging from − 45 °C in winter to 40 °C in summer^26^. Vegetation of this area is broadly segregated into alpine forests, dry alpine scrub, alpine meadows and alpine stony deserts^26^.

**Figure 1:**
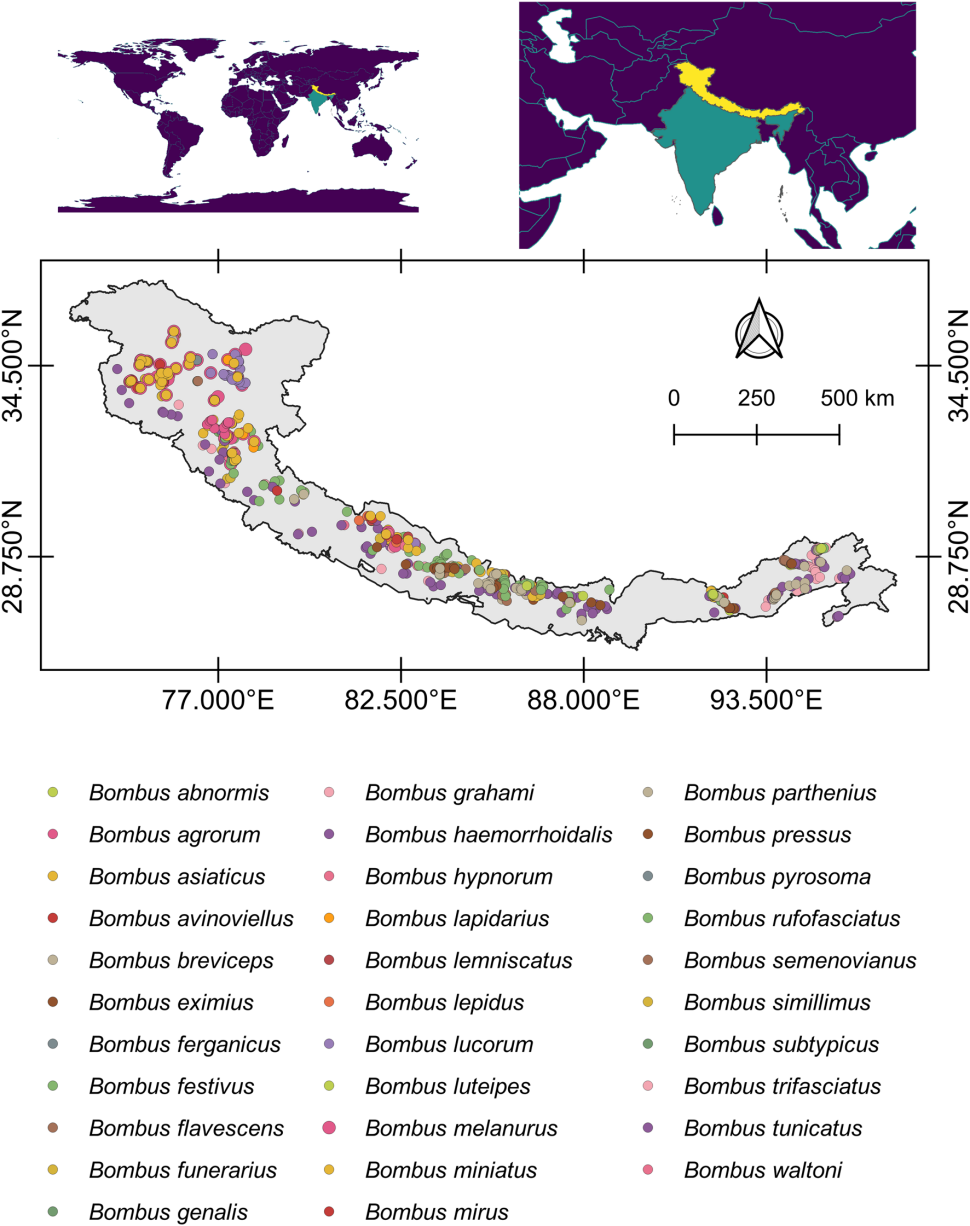
Occurrence location of 32 species of bumblebees in the Himalaya. The figure was generated using open source QGIS software version 3.28.11 (https://www.qgis.org/en/site/forusers/download.html).

We recorded the occurrence of species by opportunistic direct observation while walking slowly along roads, forest paths and hilly treks during the daytime. Since more flowering plants are found near streams that provide habitat for bumblebee foraging (especially in the trans-Himalayan region), we selected sampling sites near streams in these valleys. We used opportunistic species sighting data for the work because such data has the potential to understand signs of ecological changes, especially at a large geographic scale^27–29^. We collected 13 species (Fig. 1 of Supplementary file) by sweep netting method and identified them with the help of keys to the Indian bumblebees^30^ and submitted voucher specimens to the Department of Landscape Level Planning and Management, Wildlife Institute of India, Dehradun. We collected secondary data on the occurrence of 31 species of bumblebees (Fig. 2 of Supplementary file) in the Himalaya through the literature survey^30–35^. Though these references did not provide GPS locations for most of the species in each of the sites, they provided the names of the areas on a narrow scale (such as the name of the localities) with an elevational range. As the foraging range of bumblebees is narrow^9^, we extracted the GPS coordinates of each species for each location using Google Earth Pro. We made 1 km^2^ grid of the entire study area and removed the same species whose occurrence records fall in the same grid. In this way, we skipped two species namely *B. kashmirensis* and *B. keriensis* and kept 32 species (Fig. 1) for final analysis. For species distribution modelling (SDM) of bumblebees, we used presence-only data as it is not practical to determine the presence and absence of all species due to time and financial constraints in large mountain systems such as the Himalaya^36^.

Initially, we collected 19 bioclimatic data from https://www.worldclim.org/data/index.html, digital elevation model (DEM) data from https://www.usgs.gov/centers/eros/science/usgseros-archive-digital-elevation-shuttle-radar-topography-missionsrtm-1, land-use-land-cover (LULC) data from https://modis.gsfc.nasa.gov/data/dataprod/mod12.php, population density data from https://sedac.ciesin.columbia.edu/data/set/gpwv4-population-density-rev11, Emberger’s pluviometric quotient data and aridity index (Thornthwaite) data from https://deepblue.lib.umich.edu/data/concern/data_sets/gt54kn05f. We used single future scenario of the Shared Socioeconomic Pathway (SSPs)^37^: SSP126 (optimistic scenario designed to simulate a trajectory that is consistent with the 2 °C targets) to project the future habitat suitability of each selected species, which was collected from https://www.worldclim.org/data/cmip6/cmip6_clim30s.html. We considered 2 °C optimistic scenario because under the 2015 Paris Agreement, countries agreed to cut greenhouse gas emissions with a view to ‘holding the increase in the global average temperature to well below 2 °C above pre-industrial levels’ and due to elevation-dependent warming Asian high mountains are projected to have a higher warming of 2.1 °C ± 0.1°C^38–40^. From DEM data we calculated two data layers namely slope and aspect. Then we resampled (the process of interpolating new pixel size in raster file) all spatial data to 1 km2 and extracted the area of the Himalaya. To avoid multicollinearity between the variables we tested variance inflation factor (VIF) and retained only 13 rasters having VIF < 10^41^. These rasters were BIO 8 (Mean Temperature of Wettest Quarter), BIO 9 (Mean Temperature of Driest Quarter), BIO 15 (Precipitation Seasonality), BIO 16 (Precipitation of Wettest Quarter), BIO 17 (Precipitation of Driest Quarter), aridity index (Thornthwaite), Emberger’s pluviometric quotient, barren land, farmland, forest cover, aspect, slope and human population density.

For modelling of current and predicted future distribution of 32 species of bumblebees separately we used the Maximum Entropy (MaxEnt) algorithm^42,43^. We run the MaxEnt model by selecting 80% of the presence data to create the predictive models and the remaining 20% data to test the models^44^ and tenfold cross validation (to split the dataset into 10 subsets to calculate summary statistics, such as the area under the curve from the outputs^45^), leaving the other settings at default. In default settings the MaxEnt algorithm runs with 10,000 background points (randomly selected raster grid cells across the area of interest, which are used to determine the habitat preference of species^46,47^), 0.00001 convergence threshold (probability of the model predicting the absence of species where species is present^48^), 500 iterations (number of repeated model run until it reaches the convergence threshold^49^), 0.50 prevalence (the proportion of pixels in raster data where the algorithm assumes that the species is present for modelling purpose^50^) and logistic output format (in output maps the relative suitability ranges between 0 and 1, resulting from logistic transformation of given data based on user-specified prevalence value^51^). We used default MaxEnt settings because (1) changing MaxEnt’s default settings did not improve its performance^52^ and default settings for presence-only data achieve similar performance like tuned settings^53^, (2) it has flexibility for running even with insufficient data^51^ and (3) by default, MaxEnt algorithm assumes every pixel of the spatial data set has the same probability of being selected which tends to predict the largest possible range size consistent with the data^54^. We kept the resolution of each of the final models at 1 km^2^. We evaluated the contribution of each of the variables in each of the models by Jackknife test. We evaluated each model with the area under the curve (AUC) statistic, where AUC values closer to 1 suggest a good fit, and values closer to 0 suggest a poor fit^42^.

For the current and future species distribution, we classified the models by Jenks optimization algorithm^55^ into two categories, based on their specific optimum threshold cut-off values: 1 = unsuitable habitats, 2 = suitable habitats.

We conducted an overlap analysis among bumblebee’s habitats in the entire Himalayan region, to identify the areas that should be given priority for conservation efforts. To do this, we first overlay current habitat suitability rasters to get an overlapped map and then classified this map into three categories based on the specific optimum threshold cut off values: 1 = Non-suitable habitat, 2 = moderately suitable habitat and 3 = highly suitable habitat. We performed all analyses in Arc GIS 10.5 (Environmental Systems Research Institute, Inc., ESRI), open source QGIS version 3.28.11 and in R language and environment for statistical computing version 4.2.1^56^.

## Results

### Model accuracy

The MaxEnt SDM we made for the 32 species of bumblebees had AUC score for the training data 0.884 to 0.986 (mean 0.955 ± SD 0.024) and for the testing data 0.883 to 0.991 (mean 0.934 ± SD 0.046) which indicate high level of accuracy in the model prediction^57^.

### Predictor variable importance

**Figure 2:**
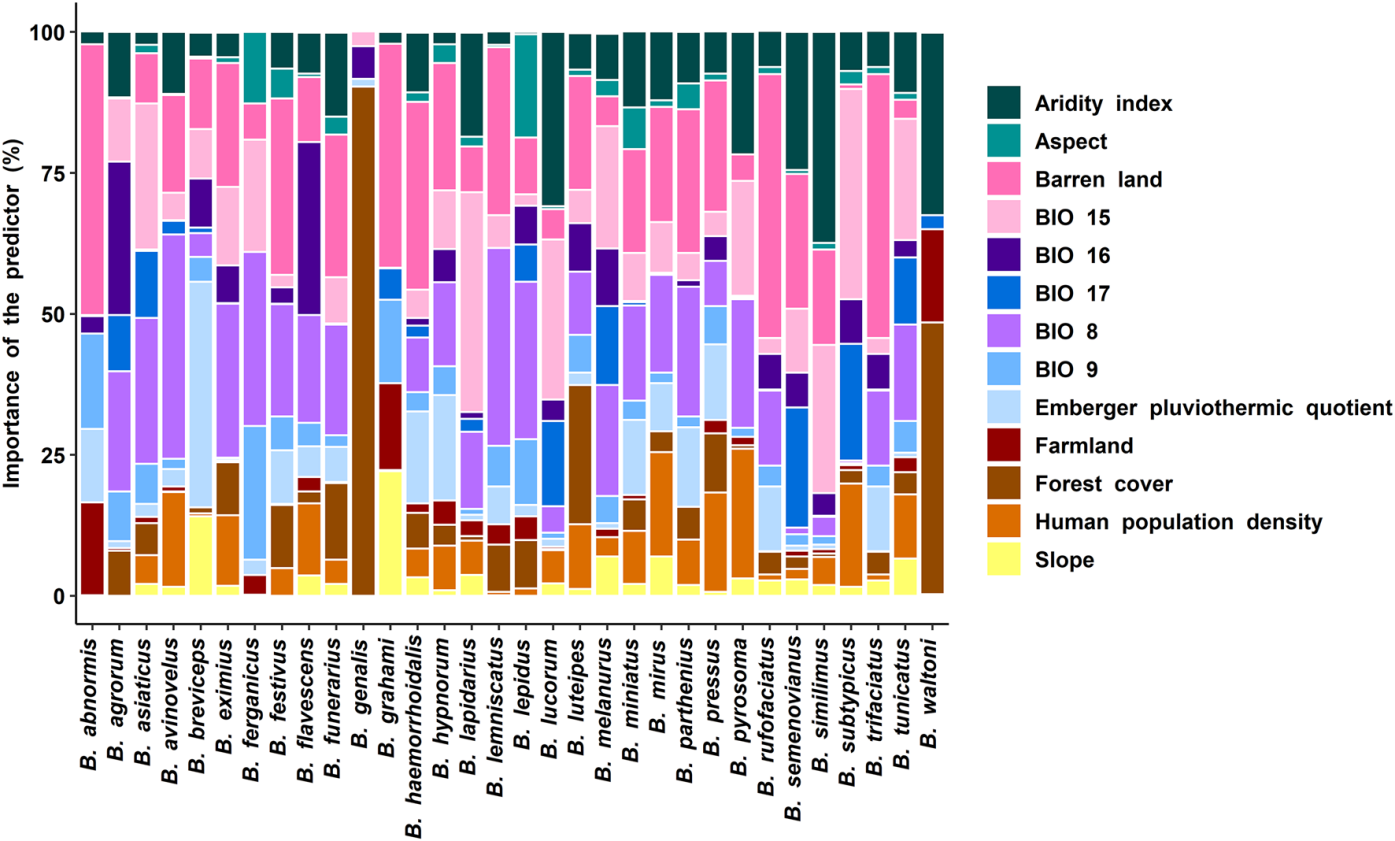
The relative importance of each variable as predictor in the MaxEnt species distribution model for different species of bumblebees in the Himalaya.

**Figure 3:**
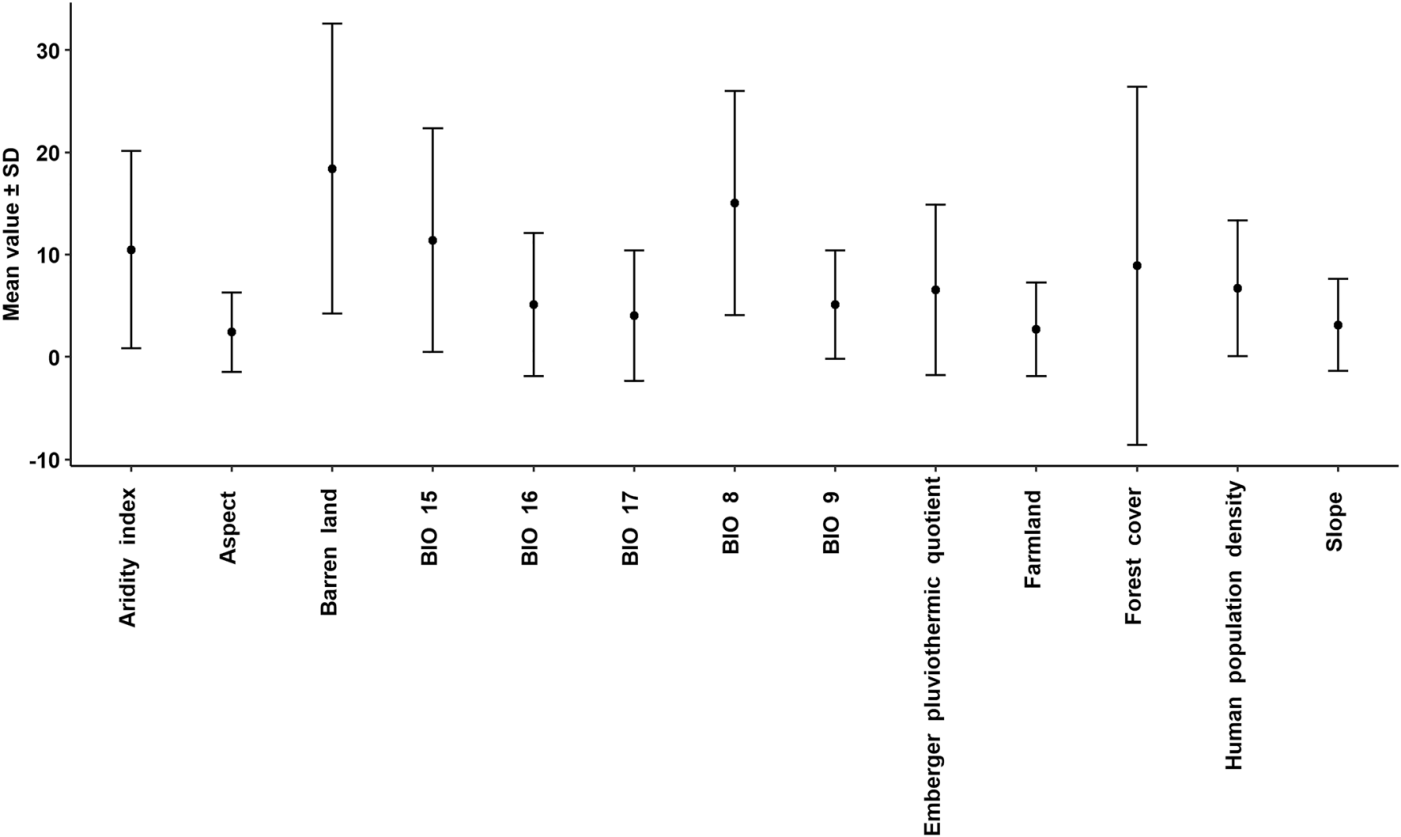
Comparative account of mean contribution of predictor variables in the MaxEnt species distribution model for different species of bumblebees in the Himalaya.

The relative importance of each variable as a predictor in the MaxEnt SDM for different species is summarized in Fig. 2. We did not find any clear pattern or species clustering when comparing the relative importance of predictor variables between species as these values varied across different species. We found that the aridity index had the highest importance (37.4%) for *B. similimus*, aspect had the highest importance (18.3%) for *B. lepidus*, barren land had the highest importance (48%) for *B. abnormis*, BIO 8 had the highest importance (39.8%) for *B. avinovelus*, BIO 9 had the highest importance (23.7%) for *B. ferganicus*, BIO 15 had the highest importance (39%) for *B. lapidarius*, BIO 16 had the highest importance (30.7%) for *B. flavescens*, BIO 17 had the highest importance (21.3%) for *B. semenovianus*, Emberger pluviothermic quotient had the highest importance (40%) for *B. breviceps*, farmland had the highest importance (16.5%) for *B. abnormis*, forest cover had the highest importance (90.3%) for *B. genalis*, human population density had the highest importance (23%) for *B. pyrosoma* and slope had the highest importance (22.1%) for *B. grahami*. If we consider the mean importance of the predictor variables, then it was observed that barren land had the highest contribution (18.4 ± SD 14.163) in bumblebee distribution in the Himalaya, followed by BIO 8 (15.053 ± SD 10.965), BIO 15 (11.406 ± SD 10.934) and aridity index (10.481 ± 9.638) (Fig. 3).

### Current suitable area

**Figure 4:**
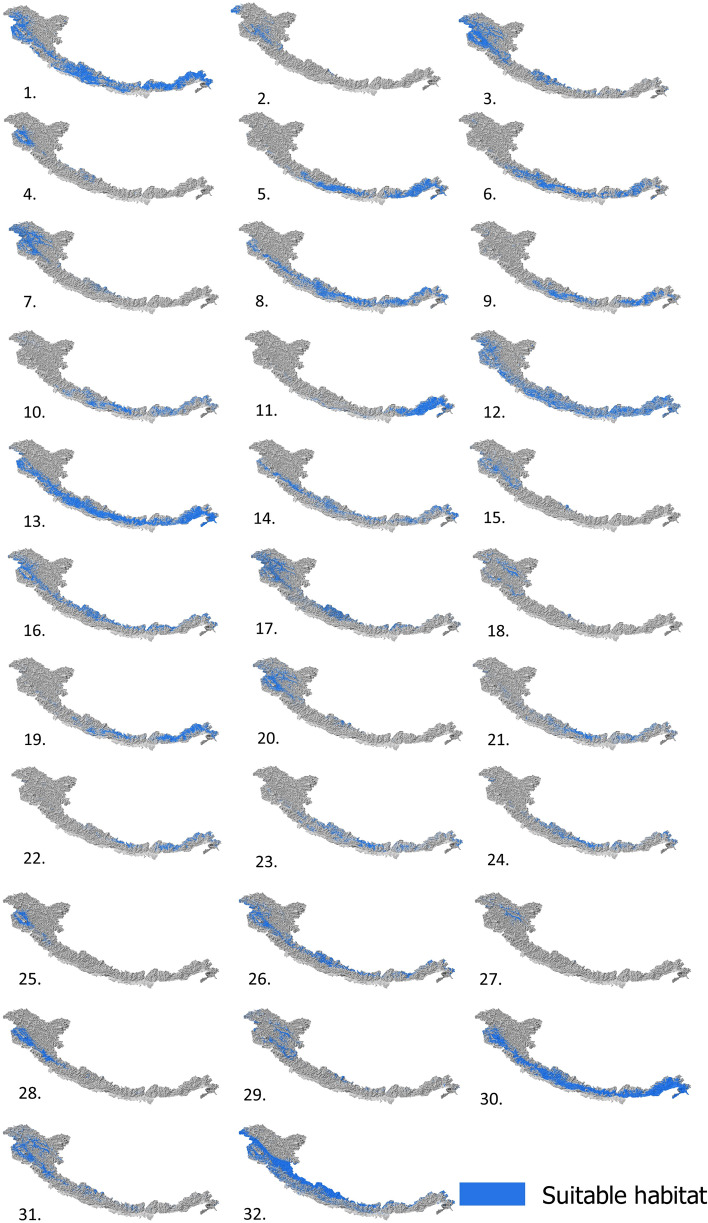
Current suitable habitat of 32 species of bumblebees in the Himalaya. (1) *B. abnormis*, (2) *B. agrorum*, (3) *B. asiaticus*, (4) *B. avinovelus*, (5) *B. breviceps*, (6) *B. eximius*, (7) *B.ferganicus*, (8) *B. festivus*, (9) *B. flavescens*, (10) *B. funerarius*, (11) *B. genalis*, (12) *B. grahami*, (13) *B. haemorrhoidalis*, (14) *B. hypnorum*, (15) *B. lapidarius,* (16) *B. lemniscatus*, (17) *B. lepidus*, (18) *B. lucorum*, (19) *B. luteipes*, (20) *B. melanurus*, (21) *B. miniatus*, (22) *B. mirus*, (23) *B. parthenius*, (24) *B. pressus*, (25) *B. pyrosoma*, (26) *B. rufofaciatus*, (27) *B. semenovianus*, (28) *B. similimus*, (29) *B. subtypicus*, (30) *B. trifaciatus*, (31) *B. tunicatus* and (32) *B. waltoni*. The figure was generated using open source QGIS software version 3.28.11 (https://www.qgis.org/en/site/forusers/download.html).

**Figure 5:**
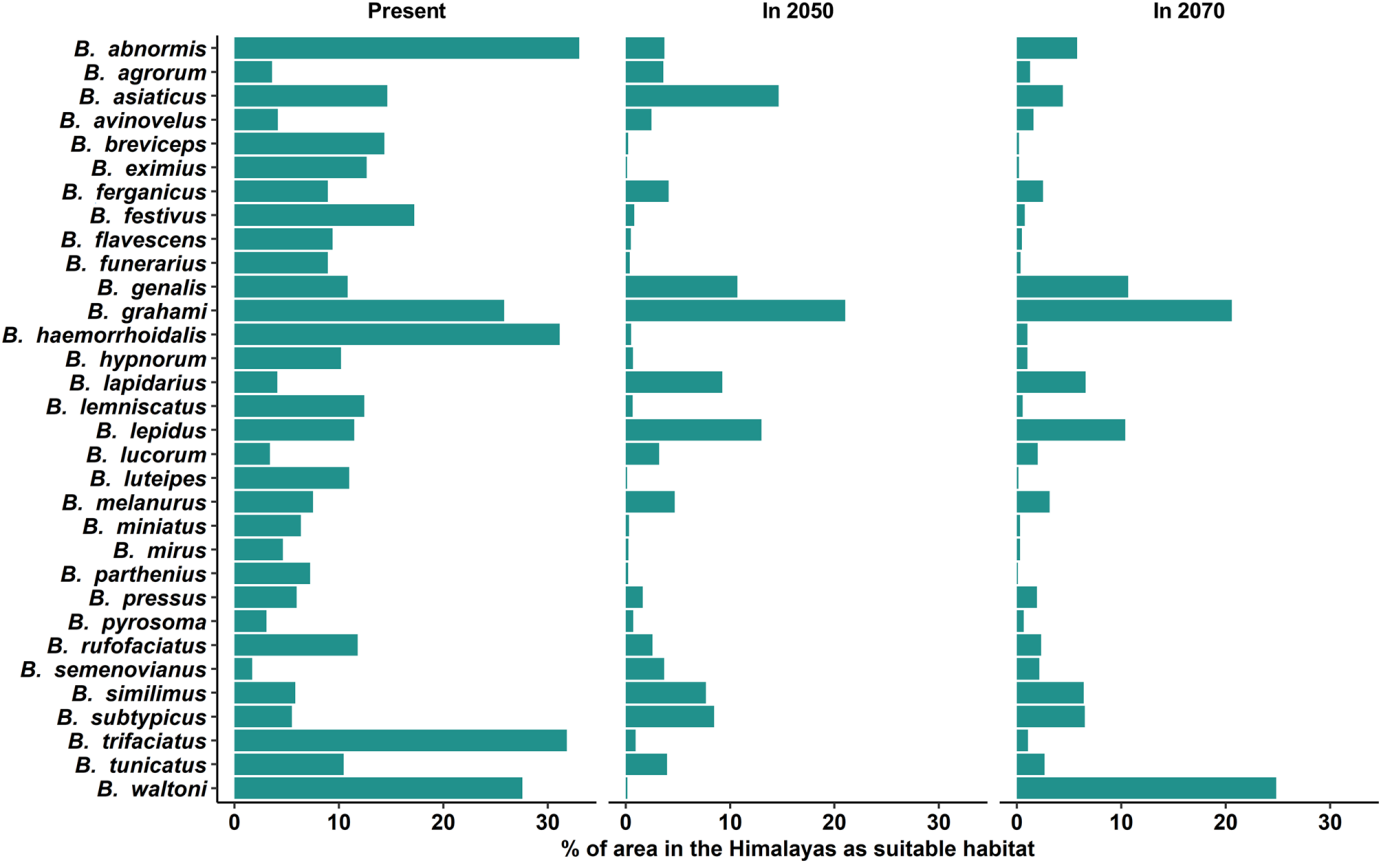
Comparative account of percentage of suitable habitat area for the bumblebees in the Himalaya for current, 2050 and 2070.

Under current climatic conditions^58^, the suitable habitats of 32 species of beetles are shown in Fig. 4. We found over 30% of the Himalayan mountain area as suitable for only three species of bumblebees namely *B. abnormis*, *B. haemorrhoidalis* and *B. trifaciatus*; 20 to 30% area as suitable for only two species of bumblebees namely *B. grahami* and *B. waltoni*; 10 to 20% area as suitable for 11 species namely *B. asiaticus*, *B. breviceps*, *B. eximius*, *B. festivus*, *B. genalis*, *B. hypnorum*, *B. lemniscatus*, *B. lepidus*, *B. luteipes*, *B. rufofaciatus* and *B. tunicatus* and less than 10% area as suitable for 16 species namely *B. agrorum*, *B. avinovelus*, *B. ferganicus*, *B. flavescens*, *B. funerarius*, *B. lapidarius*, *B. lucorum*, *B. melanurus*, *B. miniatus*, *B. mirus*, *B. parthenius*, *B. pressus*, *B. pyrosoma*, *B. semenovianus*, *B. similimus* and *B. subtypicus* out of 32 species studied (Fig. 5).

### Suitable area in 2050

**Figure 6:**
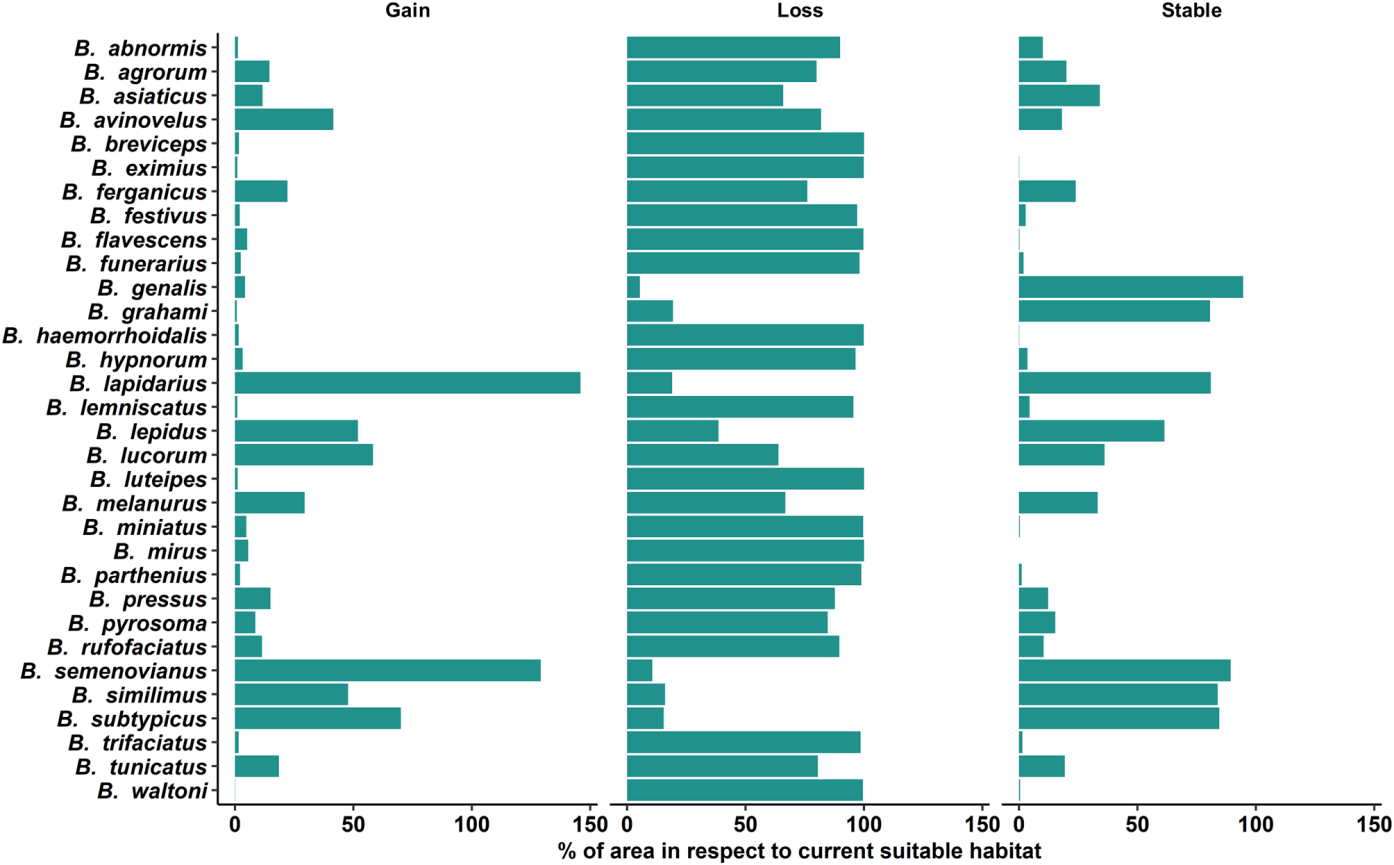
Comparative account of habitat loss, habitat gain and stable habitat in respect to current suitable habitat for the year 2050.

We found that the current suitable habitat of all bumblebee species will decrease from 5.40 to 99.99% by 2050, while their suitable habitat will increase from 0.09 to 145.80% in other regions of the Himalaya during this same period. However, decreasing or increasing suitable habitat will vary from species to species and the ratio between suitable habitat loss and suitable habitat gain will vary between 0.08 and 1090.15. We observed that 15 species will lose more than 90% of their current suitable habitat and 10 species will lose more than 50–90% of their current suitable habitat. Among these 25 species, only *B. lucorum* will lose about 63.87% of its current habitat but it will also get about 58.26% new suitable habitats. About 94.59% of the current suitable habitat of *B. genalis* will be stable. If we consider the quantity of suitable habitat concerning the total area of the Himalaya, we found that in 2050 more than 20% of the area of the Himalaya will be suitable for only one species (*B. grahami*). From 10 to 20% of the area of the Himalaya will be suitable for only three species namely *B. asiaticus*, *B. genalis* and *B. lepidus*. Five species will experience an increase in their suitable habitat areas namely *B. lapidarius*, *B. lepidus*, *B. semenovianu*s, *B. similimus* and *B. subtypicus*. More than 100% of suitable habitat will increase for *B. lapidarius* and *B. semenovianus*. The percentage of suitable habitat area for the bumblebees in the Himalaya for the year 2050 is shown in Fig. 5. Comparative account of habitat loss, habitat gain and stable habitat concerning current suitable habitat for the year 2050 is shown in Fig. 6. See Figs. 3–18 of the Supplementary file for maps of habitat suitability changes for Himalayan bumblebees in 2050.

### Suitable area in 2070

**Figure 7:**
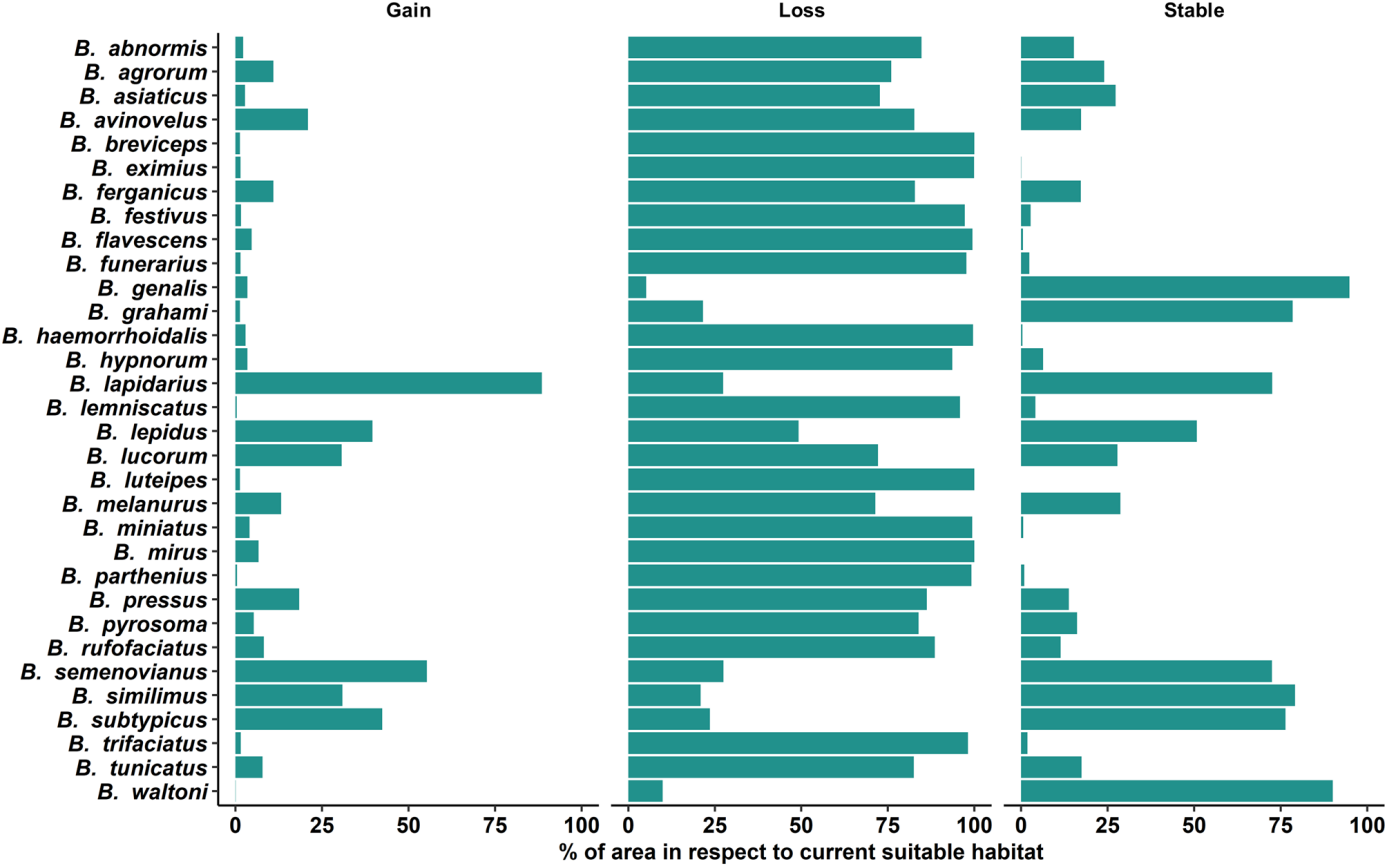
Comparative account of habitat loss, habitat gain and stable habitat in respect to current suitable habitat for the year 2070.

We found that the current suitable habitat of all bumblebee species will decrease from 5.13 to 100% by 2070, while their suitable habitat will increase from 0.04 to 88.48% in other regions of the Himalaya during this same period. However, decreasing or increasing suitable habitat will vary from species to species and the ratio between suitable habitat loss and suitable habitat gain will vary between 0.31 and 247.75. We observed that 13 species will lose more than 90% of their current suitable habitat and 11 species will lose more than 50–90% of their current suitable habitat. Among these 24 species, only *B. lepidus* will lose about 49.19% of its current habitat but it will also get about 39.58% new suitable habitat. About 94.87% of the current suitable habitat of *B. genalis* will be stable. If we consider the quantity of suitable habitat concerning the total area of the Himalaya, we found that in 2070 more than 20% of the area of the Himalaya will be suitable for only two species (*B. grahami* and *B. waltoni*). Above 10% of the area will be suitable for only two species *B. lepidus* and *B. genalis*. Four species will experience an increase in their suitable habitat areas namely *B. lapidarius*, *B. semenovianus*, *B. subtypicus* and *B. similimus*. Among these species, *B. lapidarius* will experience increment of suitable area above 60%. The percentage of suitable habitat area for the bumblebees in the Himalaya for the year 2070 is shown in Fig. 5. Comparative account of habitat loss, habitat gain and stable habitat in respect to current suitable habitat for the year 2070 is shown in Fig. 7. See Figs. 3–18 of the Supplementary file for maps of habitat suitability changes for Himalayan bumblebees in 2070.

### Conservation priority area

**Figure 8:**
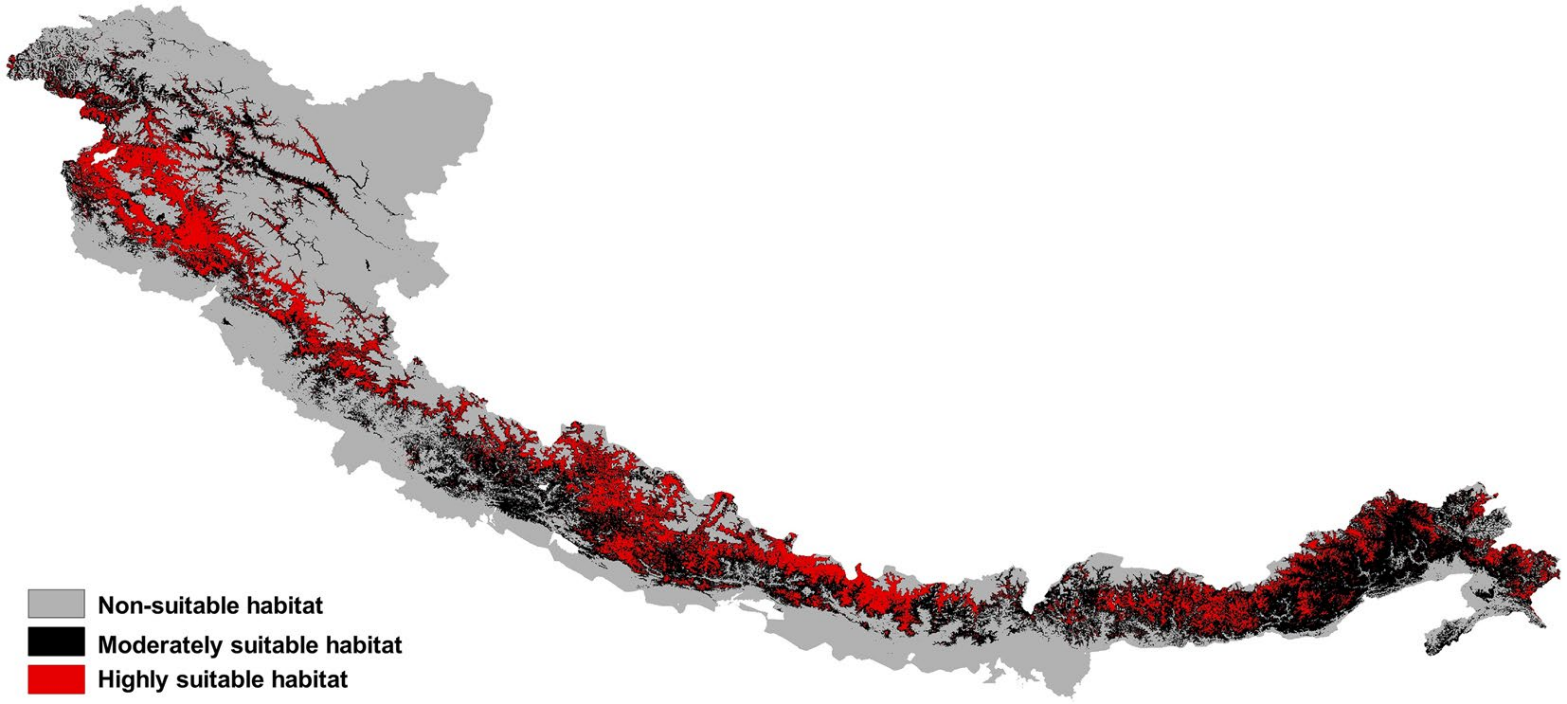
Habitat suitability of bumblebees in the Himalaya. The area marked in the red colour should be considered as ‘conservation priority area’ for bumblebees in the Himalaya. The figure was generated using open source QGIS software version 3.28.11 (https://www.qgis.org/en/site/forusers/download.html).

By overlaying current suitable habitat of all 32 bumblebee species, we found that 15.82% of the area had highly suitable habitat for them, followed by 28.04% moderately suitable and 55.62% less suitable (Fig. 8). These highly suitable habitat areas were mainly observed in the Western Himalaya (in Dhauladhar range of Himachal Pradesh and Pir Panjal range of Jammu and Kashmir and Himachal Pradesh). We also observed some highly suitable habitat patches in the Central Himalaya and Eastern Himalaya. We considered these areas with highly suitable habitats for bumblebees as conservation priority areas where appropriate measures should be taken to protect bumblebees.

## Discussion

No single predictor variable made the largest contribution for all bumblebee species, and different predictor variables were differentially important for each species, showing us that the ecological niche of these temperate pollinators is diversified across their Himalayan range. However, statistically, the LULC and bioclimatic variables mainly influenced their distribution. We find that barren land, BIO 8, BIO 15 and aridity index were the most important predictors of their distribution. Ecosystem aridity can directly and indirectly affect the distribution of pollinating insects. If influenced directly, bees may suffer from desiccation stress due to increasing drought, which can have detrimental effects on their population38. Because increasing drought can reduce plant diversity that can regulate plant-pollinator interactions, drought can also have indirect effects on pollinator communities^59^. Particularly in mountainous regions, the frequency and diversity of bumblebees are influenced by drought^60^. In this study, we also found that the barren land and drought had an impact on the distribution of bumblebees in the Himalayas. The temperature of the rainy season can influence the distribution of bumblebees^61–63^. We found that the average temperature of the wettest district had a major impact on the distribution of bumblebees in the Himalayas. We found that precipitation seasonality (BIO 15) has a strong influence on bumblebee distribution and this finding is similar to the results of other studies^23,64,65^ which also observed that bumblebee occurrence and abundance are influenced by precipitation seasonality. Since bumblebee populations are sensitive to the timing of resource availability^66^, it can be concluded that fluctuations in temperature and precipitation influences snowfall, snow melt, and precipitation regimes which in turn affect habitat conditions by influencing nectar availability for pollinators^67–69^.

The image of the future state of the Himalayan bumblebees we found in our study is a profound concern. This is because the amount of suitable habitat for most of them will be reduced in both 2050 and 2070. The SDM estimated that by 2050, more than 75% of species will experience habitat loss, among them more than 40% will experience a loss of 90% of their habitat and above 45% of species will have less than 1% of the area as suitable habitat. There is no sign of improvement in 2070 as in this period, SDM estimated that more than 85% of species will experience habitat loss, among them more than 40% will experience loss of 90% of their habitat and above 35% of species will have less than 1% area as suitable habitat. Although the models predict future declines in overall bumblebee habitat, not all bumblebee species will respond in the same way to climate change impacts. We observed that while most species of bumblebee habitat will decline significantly in the future, for some species it will not decline, but rather remain stable or increase. Such disparate effects of climate change on bumblebee habitats may be due to their specialised life history traits such as habitat preference, foraging range, feeding adaptations, nesting behaviour and body size^9,70,71^. In this study, we observed that more than 90% of the current suitable habitat of *B. genalis* will remain suitable in 2050 and 2070 and the suitable habitat area for four species (*B. lapidarius*, *B. semenovianus*, *B. similimus* and *B. subtypicus*) would increase in both 2050 and 2070 rather than decrease, and even for some species this increased value is more than 100%. This finding is consistent with the fact that highly mobile, super-generalists, short-tongued bumblebees *B. terrestris* and *B. lapidarius* becoming increasingly dominant in Europe probably due to climate change^72–75^. Climate change and anthropogenic activities such as habitat fragmentation by deforestation affect differentially in different bumblebee species, based on their life history traits. But species-specific life history traits are still unknown for Himalayan bumblebee species. To fill this knowledge gap, species-specific life history studies should be carried out for proper conservation of these precious pollinators.

Prioritization of target areas and species is important for effective planning of biodiversity protection. Studies found that in Asian countries bumblebees became vulnerable to climate and land cover changes^76^. The role of protected areas (PAs) in wildlife conservation is generally considered to be necessary. Though the PAs are generally designed for vertebrates and plants but they can also provide undisturbed habitat for insects also^77^. However, approximately 76% of all insect species documented in the Global Biodiversity Information Facility (GBIF) and having at least three occurrence records globally do not receive conservation benefits from PAs because, in most cases, their habitats are located outside the jurisdiction of the PAs^78^. Thus bumblebees and other ecologically important insects should be explicitly included in the conservation management plans to stop the decline of the insect population. But at the same time, we should keep in mind that insufficient management can lead to the decline of the insect pollinator population even inside the PAs^79^. In this work, we found that only about 15% of the total area of the Himalaya can be considered a conservation priority area for bumblebees. But still, we do not have any reliable information about how many of these suitable habitats fall within the jurisdiction of Himalayan PAs across different countries. We recommended that further studies should be carried out to understand if any spatial mismatches exist between existing PAs and conservation priority areas for the bumblebees in the Himalaya. As the current suitable habitat within PAs will become unsuitable for the insects due to climate change^80^, the extent and boundaries of current PAs in the Himalaya should be redefined to accommodate changes in suitable habitat over time.

The assessment by the Intergovernmental Science-Policy Platform on Biodiversity and Ecosystem Services (IPBES) admitted that the ranges, abundances and seasonal activities of some wild pollinator species like bumblebees have changed in response to observed climate change over recent decades^81^. Shortly after this assessment was published, member states of the Convention on Biological Diversity (CBD) were making commitments to support pollinator conservation, and some countries formulated national pollinator strategies and action plans^82^. But studies on trends and the impact of the pollinator decline are concentrated in high-income countries in North America and Europe, and almost absent in regions which are thought to be most vulnerable to pollinator diversity^83^. The Himalaya is such a vulnerable region where the shrinking of the cryosphere, land use change, vegetation change and loss of biodiversity have adversely affected the ecosystem services^84^. Our study indicates that due to the impact of the changing climate, the suitable habitat of the majority of the bumblebees will be curtailed in the next 50 years. This finding is consistent with earlier studies^85–87^ which predicted that due to anthropogenic activities and climate change, the dense forest cover will be drastically reduced to only 10% by the year 2100, which will lead to a broad range of biodiversity on the verge of extinction. It is anticipated that global climate change mitigation policies can play an effective role in bumblebee conservation^88^. Although the countries of Afghanistan, Pakistan, India, China, Nepal, Bhutan and Myanmar share international borders in the Himalayan region, there has been an absence of any international agreement for comprehensive pollinator diversity conservation initiatives in the region so far. Naturally, we have observed that the suitable habitat and conservation priority areas of bumblebees do not obey any international boundary. So these countries that share international borders and natural habitats of bees through the Himalaya should develop policies to restore and protect transboundary natural habitats for these pollinators as well as other wild species. In fact, common interest in biodiversity conservation could be part of diplomatic toolkit between Himalayan countries^89^.

There are six stages^90^ present for the development and implementation of biodiversity conservation and climate mitigation targets namely (1) field observations and simulations (2) empirical generalizations (3) negotiated choice of preferred outcome (4) politically informed interpretation of target (5) implementation actions and (6) monitoring and adaptive management. Our work fulfils the first two criteria where using both field observations and SDM simulations we showed that most bumblebee species of Himalaya are likely to be affected significantly by climate change. But to minimize the impact of climate change rest four stages should also be met. As biodiversity conservation in PAs is contingent on their location, management and governance^90^ the government should make adequate pollinator conservation policy in PAs in line with international agreements and goals. Since 1981 Government of India running the “All India Coordinated Research Project on Honey Bees and Pollinators” through the Indian Council of Agricultural Research (ICAR) for the conservation of pollinators to improve farmer’s livelihoods and enhance ecosystem services. But the scheme was mainly for the conservation of agro-ecosystem pollinators and not much emphasis was given to forest pollinators. About 200 nations committed to protect 30% of the Earth as protected area by 2030 under the United Nations (UN) Convention on Biological Diversity’s (CBD) Kunming-Montreal Global Biodiversity Framework^91^. Though an ecologist can identify a problem and provide the solution to that problem but implementing the series of operational steps to mitigate the problem through the projects can be done by project managers and policy makers^92^. Thus, the catastrophic effects of climate change leading to the loss of Himalayan pollinators can be mitigated by adopting science-based and policy-supported approaches that will preserve their natural habitats in the region.

### Supplementary Information

Supplementary Figures.

## Data Availability

The data that support the findings of this study are available from the corresponding author upon reasonable request.
