# Supplementary material for: Unveiling of climate change-driven decline of suitable habitat for Himalayan bumblebees
Source: Sci Rep. 2024 Feb 29;14:4983. doi: 10.1038/s41598-024-52340-9 (PMC10904386; doi:10.1038/s41598-024-52340-9)
Supplement: Supplementary file 1 — Supplementary Figures. [file 41598_2024_52340_MOESM1_ESM.pdf]

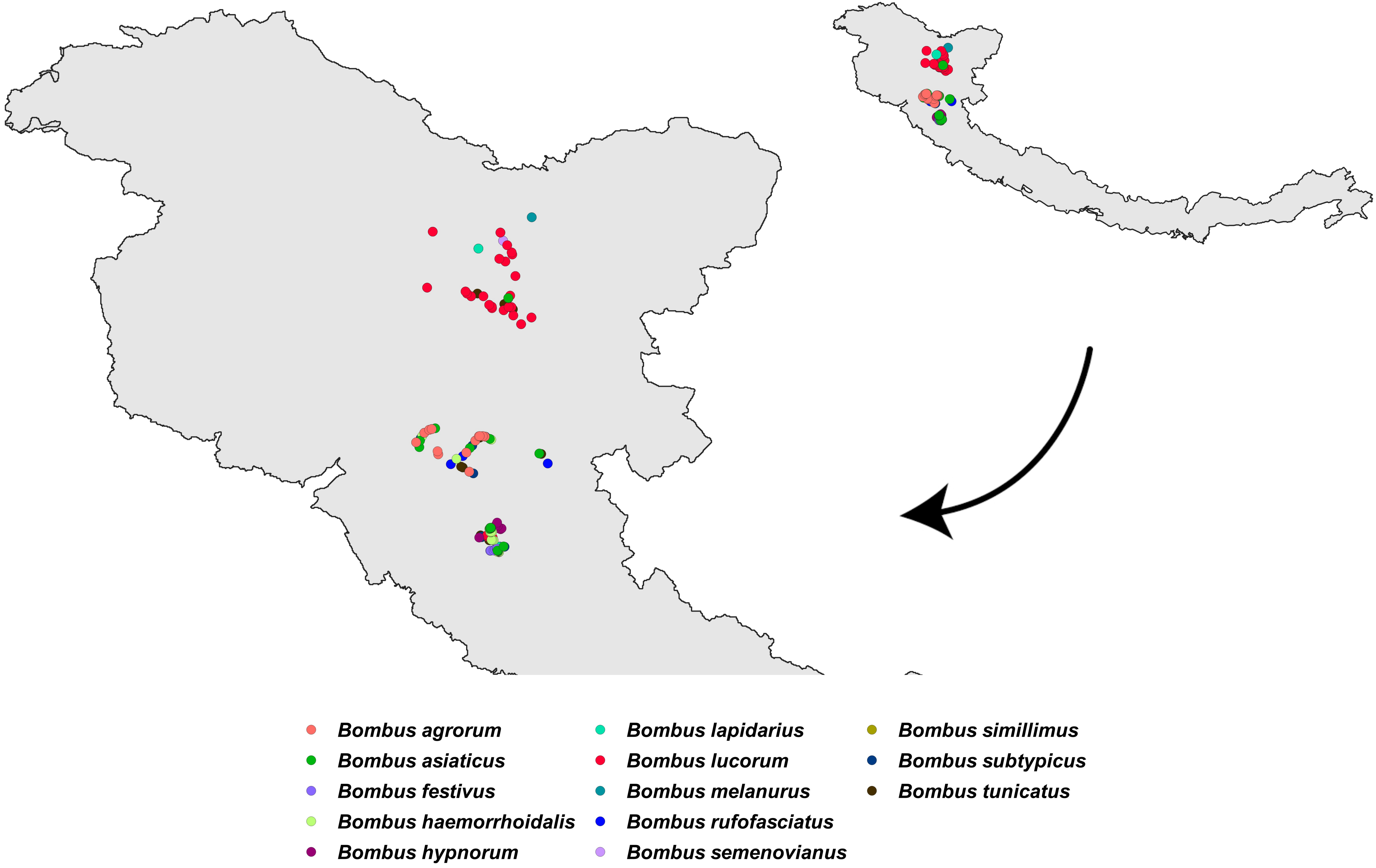

Supplementary figure 1: Occurrence location of 13 species of bumblebees in Western Himalaya and Trans-Himalaya based on field survey. The figure was generated using open source QGIS software version 3.28.11 (https://www.qgis.org/en/site/forusers/download.html).

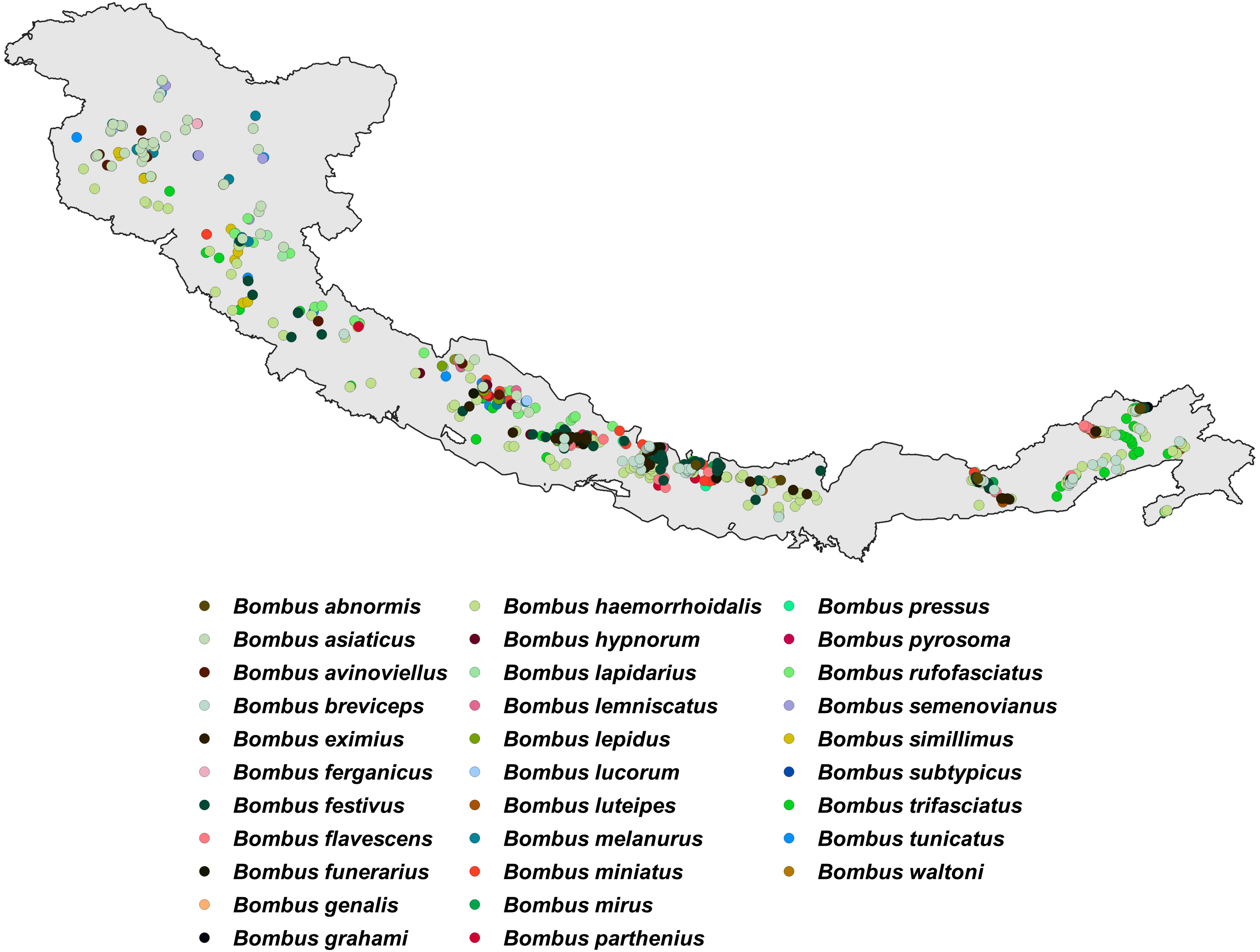

Supplementary figure 2: Occurrence location of 31 species of bumblebees in Himalaya based on literature survey. The figure was generated using open source QGIS software version 3.28.11 (https://www.qgis.org/en/site/forusers/download.html).

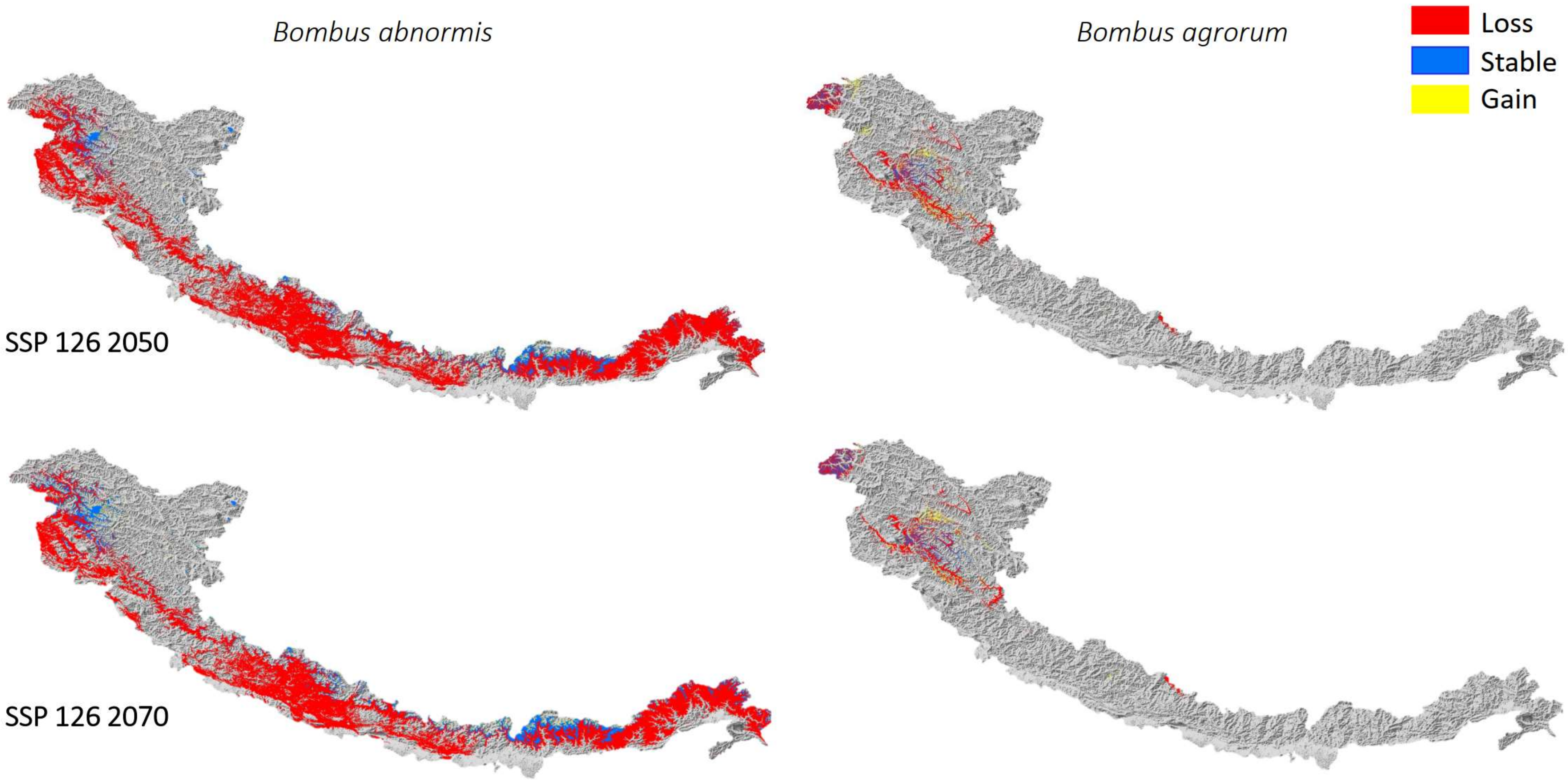

Supplementary figure 3: Predicted future (in 2050 and 2070) habitat suitability of *Bombus abnormis* and *Bombus agrorum* in the Himalaya. The figure was generated using open source QGIS software version 3.28.11 (https://www.qgis.org/en/site/forusers/download.html).

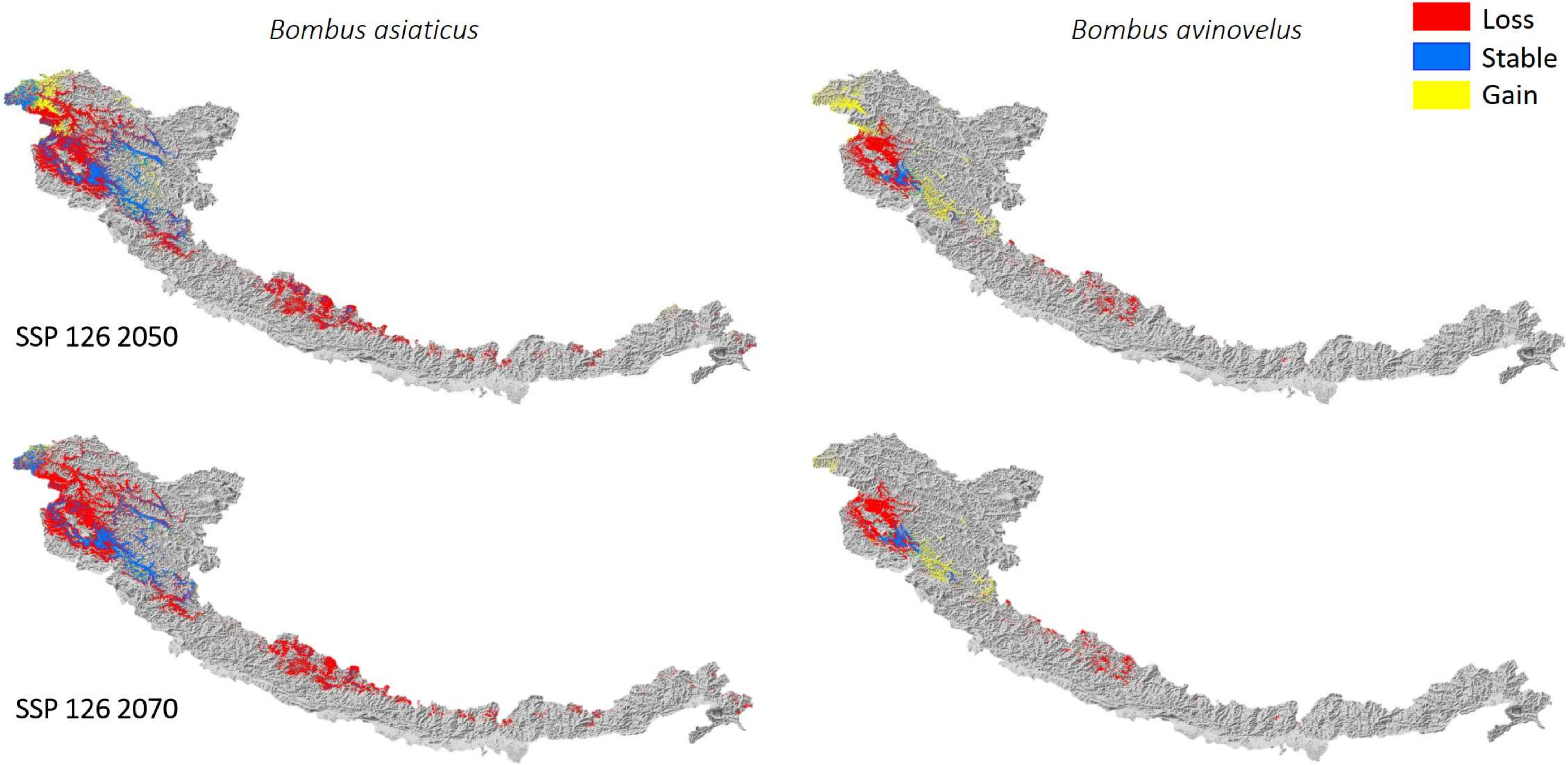

Supplementary figure 4: Predicted future (in 2050 and 2070) habitat suitability of *Bombus asiaticus* and *Bombus avinovelus* in the Himalaya. The figure was generated using open source QGIS software version 3.28.11 (https://www.qgis.org/en/site/forusers/download.html).

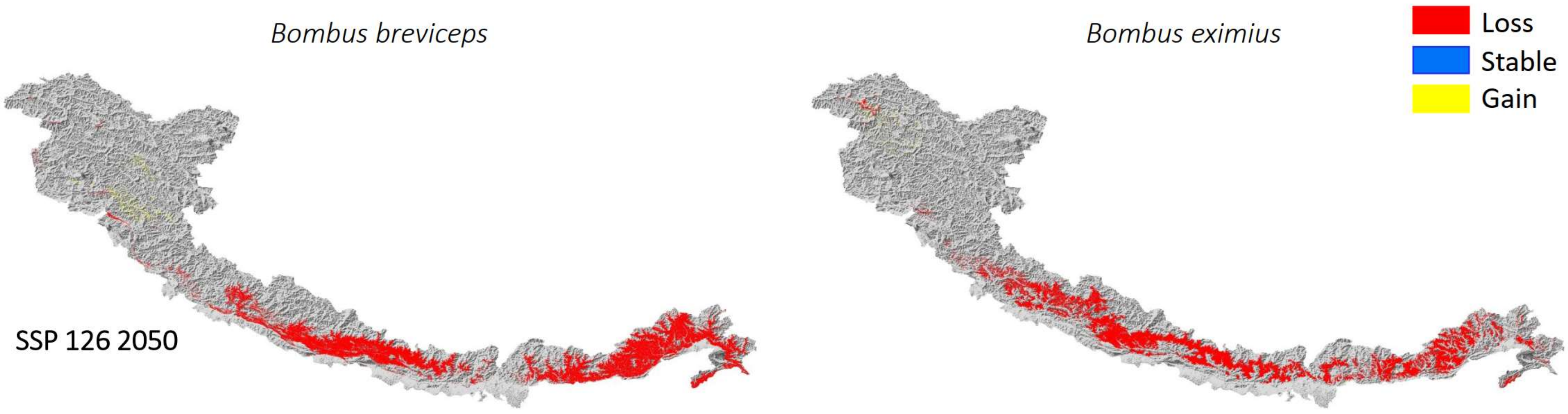

Supplementary figure 5: Predicted future (in 2050 and 2070) habitat suitability of *Bombus breviceps* and *Bombus eximius* in the Himalaya. The figure was generated using open source QGIS software version 3.28.11 (https://www.qgis.org/en/site/forusers/download.html).

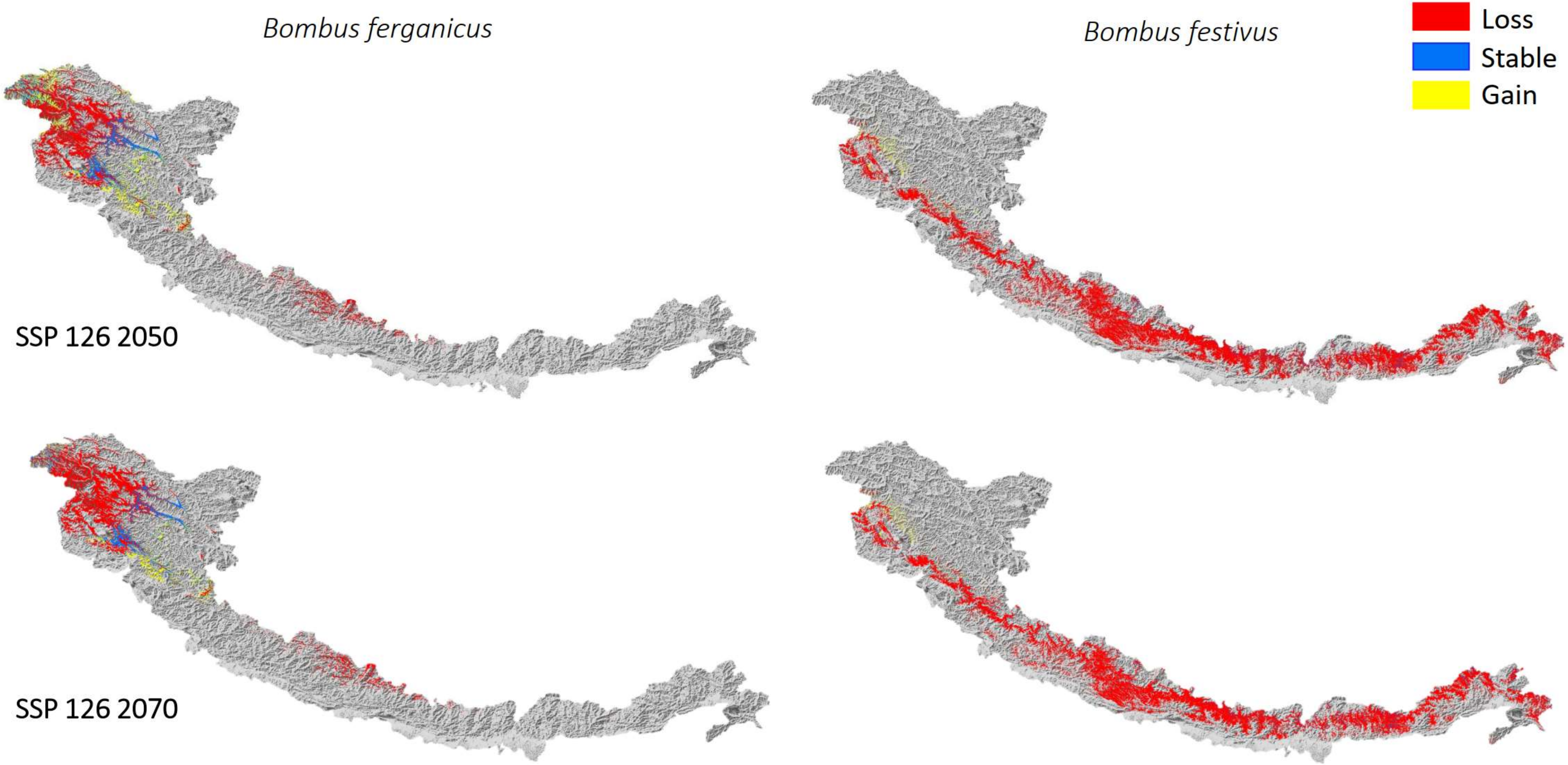

Supplementary figure 6: Predicted future (in 2050 and 2070) habitat suitability of *Bombus ferganicus* and *Bombus festivus* in the Himalaya. The figure was generated using open source QGIS software version 3.28.11 (https://www.qgis.org/en/site/forusers/download.html).

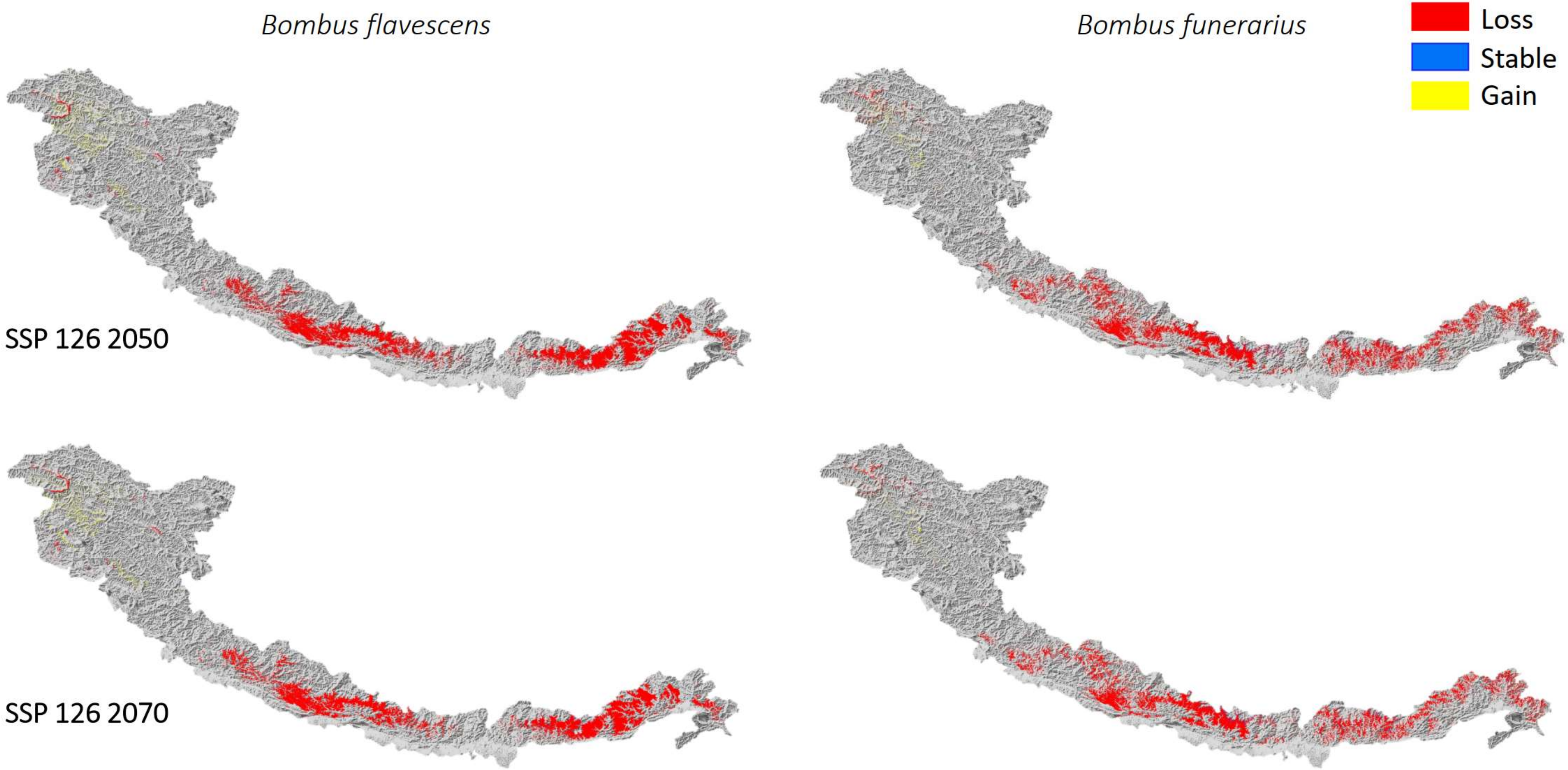

Supplementary figure 7: Predicted future (in 2050 and 2070) habitat suitability of *Bombus flavescens* and *Bombus funerarius* in the Himalaya. The figure was generated using open source QGIS software version 3.28.11 (https://www.qgis.org/en/site/forusers/download.html).

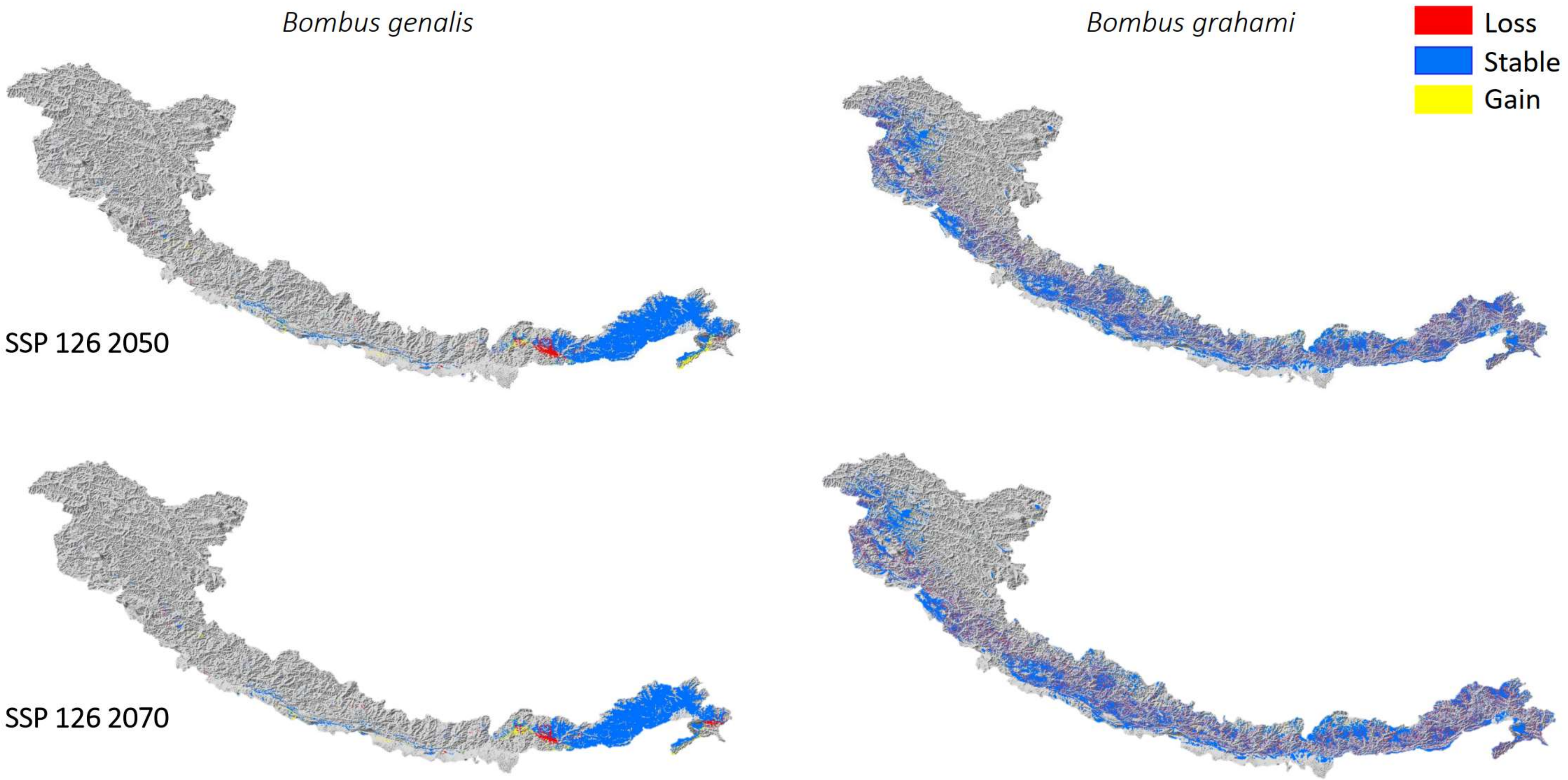

Supplementary figure 8: Predicted future (in 2050 and 2070) habitat suitability of *Bombus genalis* and *Bombus grahami* in the Himalaya. The figure was generated using open source QGIS software version 3.28.11 (https://www.qgis.org/en/site/forusers/download.html).

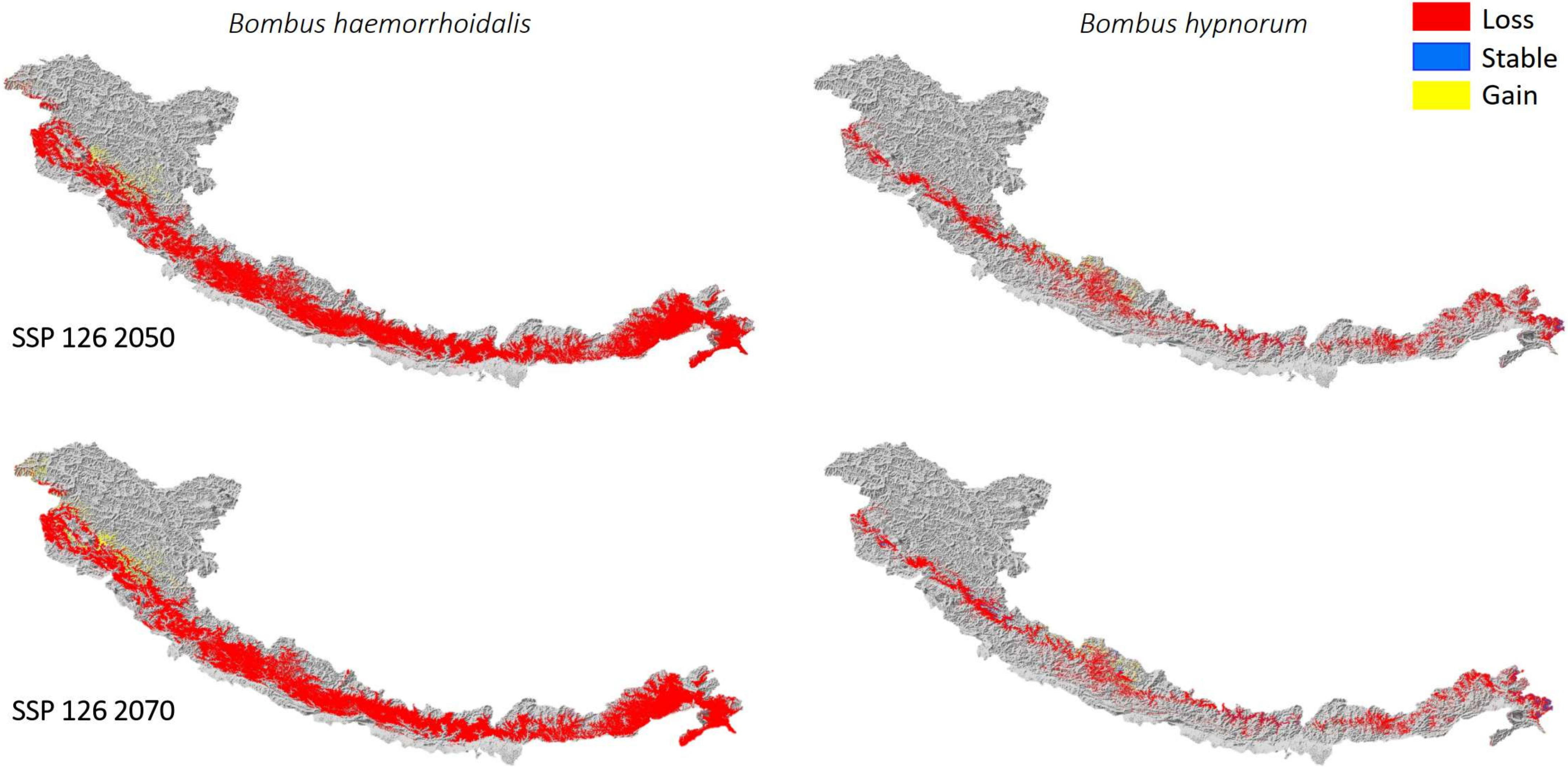

Supplementary figure 9: Predicted future (in 2050 and 2070) habitat suitability of *Bombus haemorrhoidalis* and *Bombus hypnorum* in the Himalaya. The figure was generated using open source QGIS software version 3.28.11 (https://www.qgis.org/en/site/forusers/download.html).

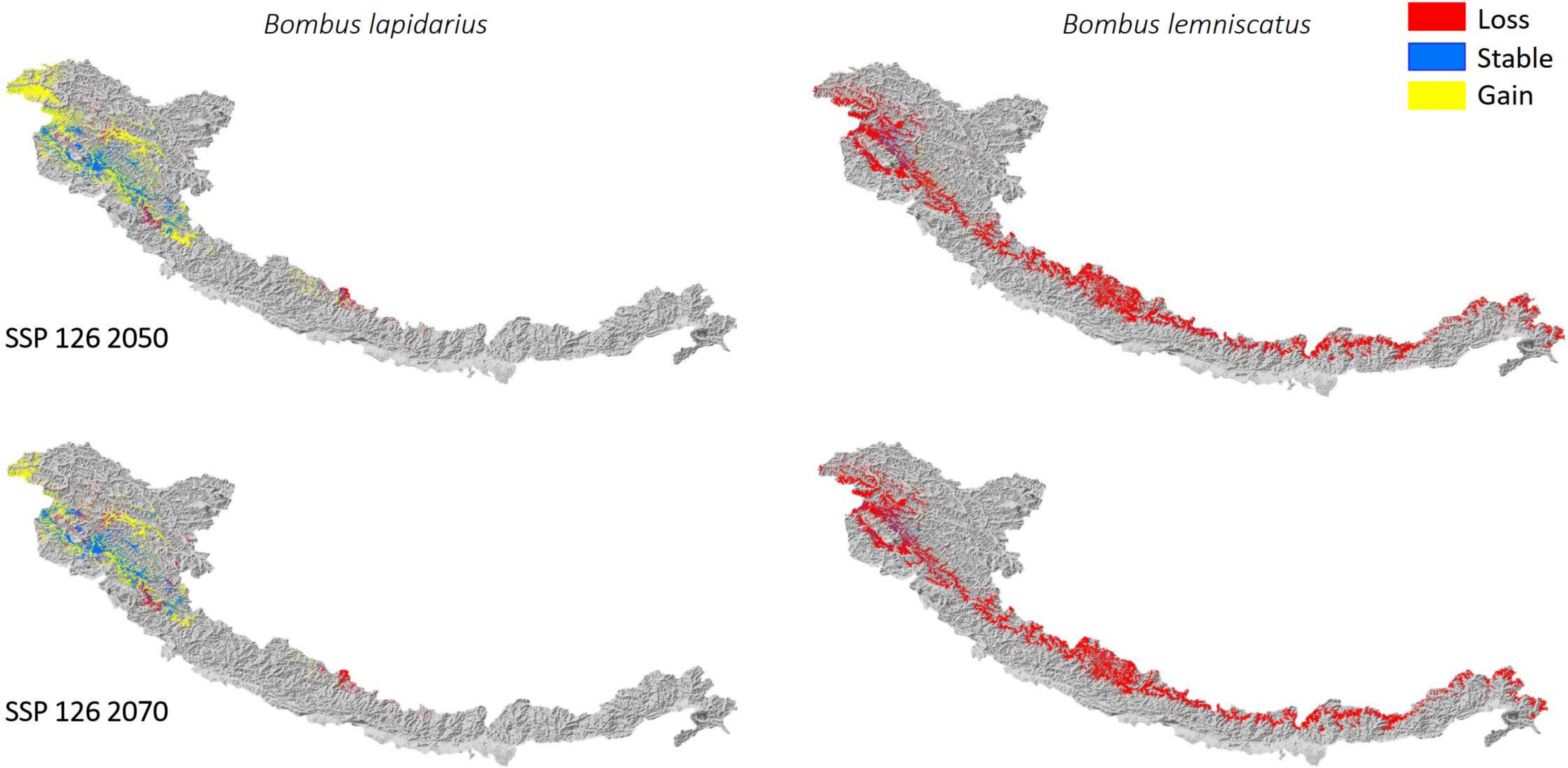

Supplementary figure 10: Predicted future (in 2050 and 2070) habitat suitability of *Bombus lapidarius* and *Bombus lemniscatus* in the Himalaya. The figure was generated using open source QGIS software version 3.28.11 (https://www.qgis.org/en/site/forusers/download.html).

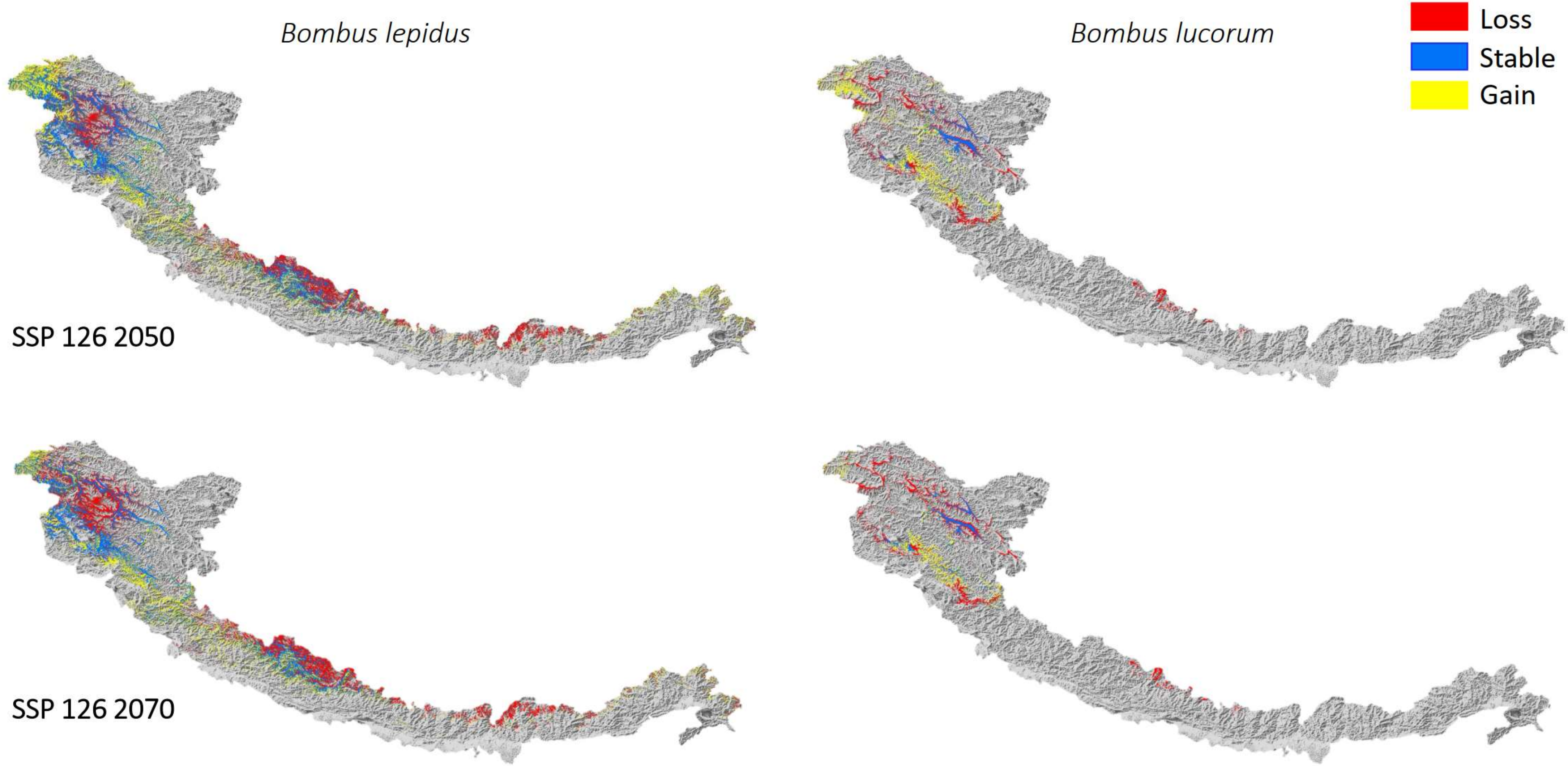

Supplementary figure 11: Predicted future (in 2050 and 2070) habitat suitability of *Bombus lepidus* and *Bombus lucorum* in the Himalaya. The figure was generated using open source QGIS software version 3.28.11 (https://www.qgis.org/en/site/forusers/download.html).

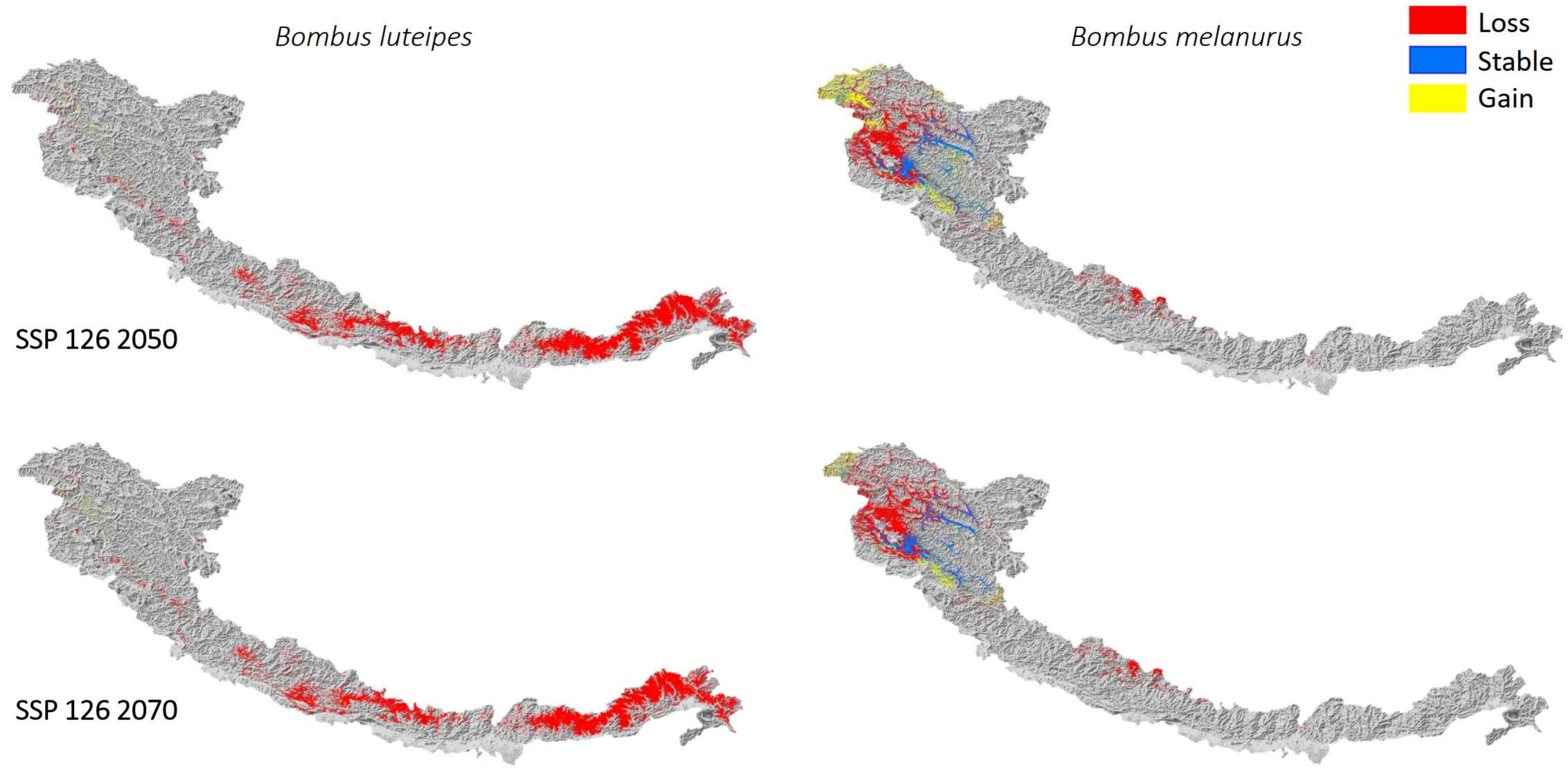

Supplementary figure 12: Predicted future (in 2050 and 2070) habitat suitability of *Bombus luteipes* and *Bombus melanurus* in the Himalaya. The figure was generated using open source QGIS software version 3.28.11 (https://www.qgis.org/en/site/forusers/download.html).

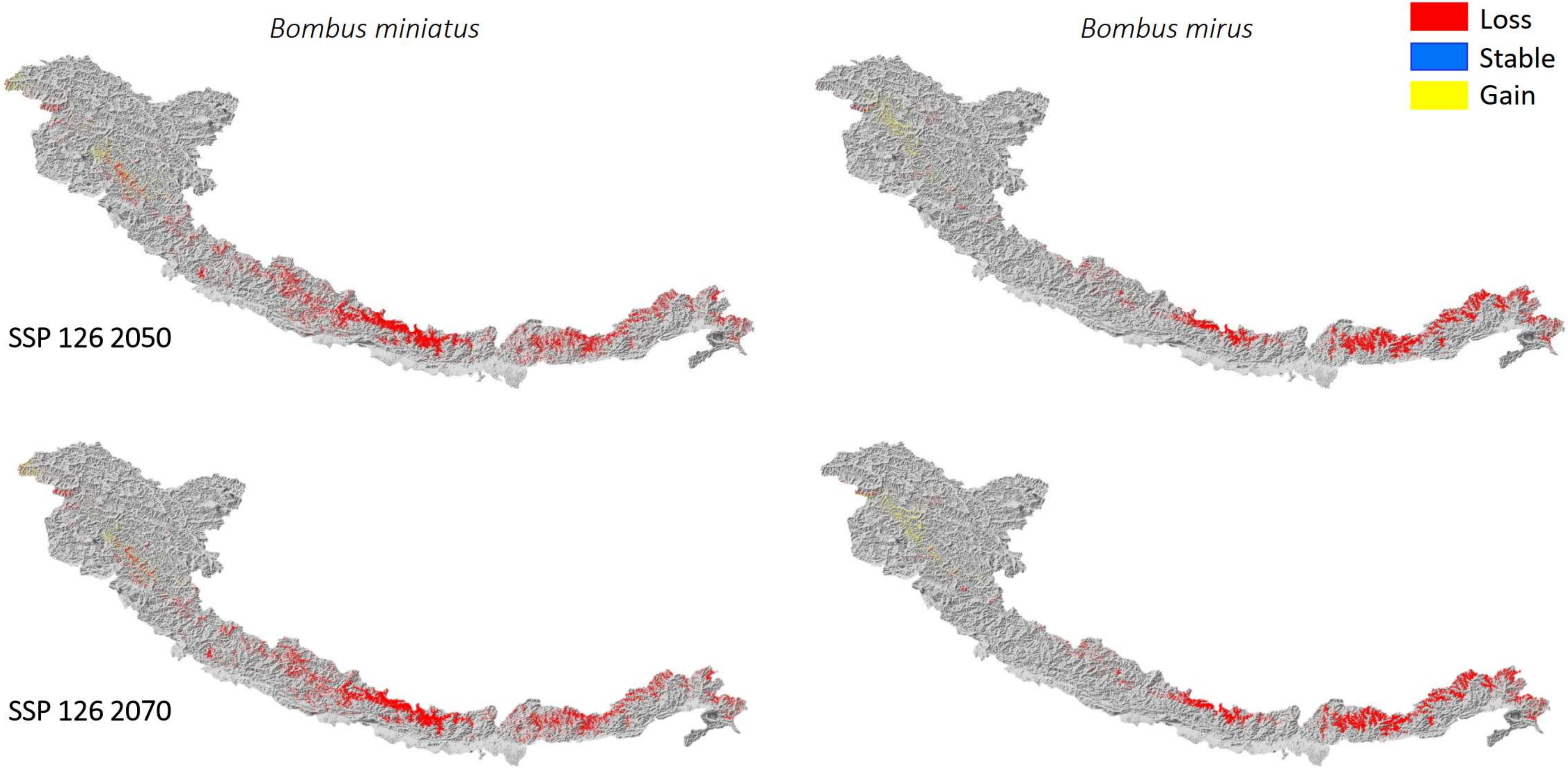

Supplementary figure 13: Predicted future (in 2050 and 2070) habitat suitability of *Bombus miniatus* and *Bombus mirus* in the Himalaya. The figure was generated using open source QGIS software version 3.28.11 (https://www.qgis.org/en/site/forusers/download.html).

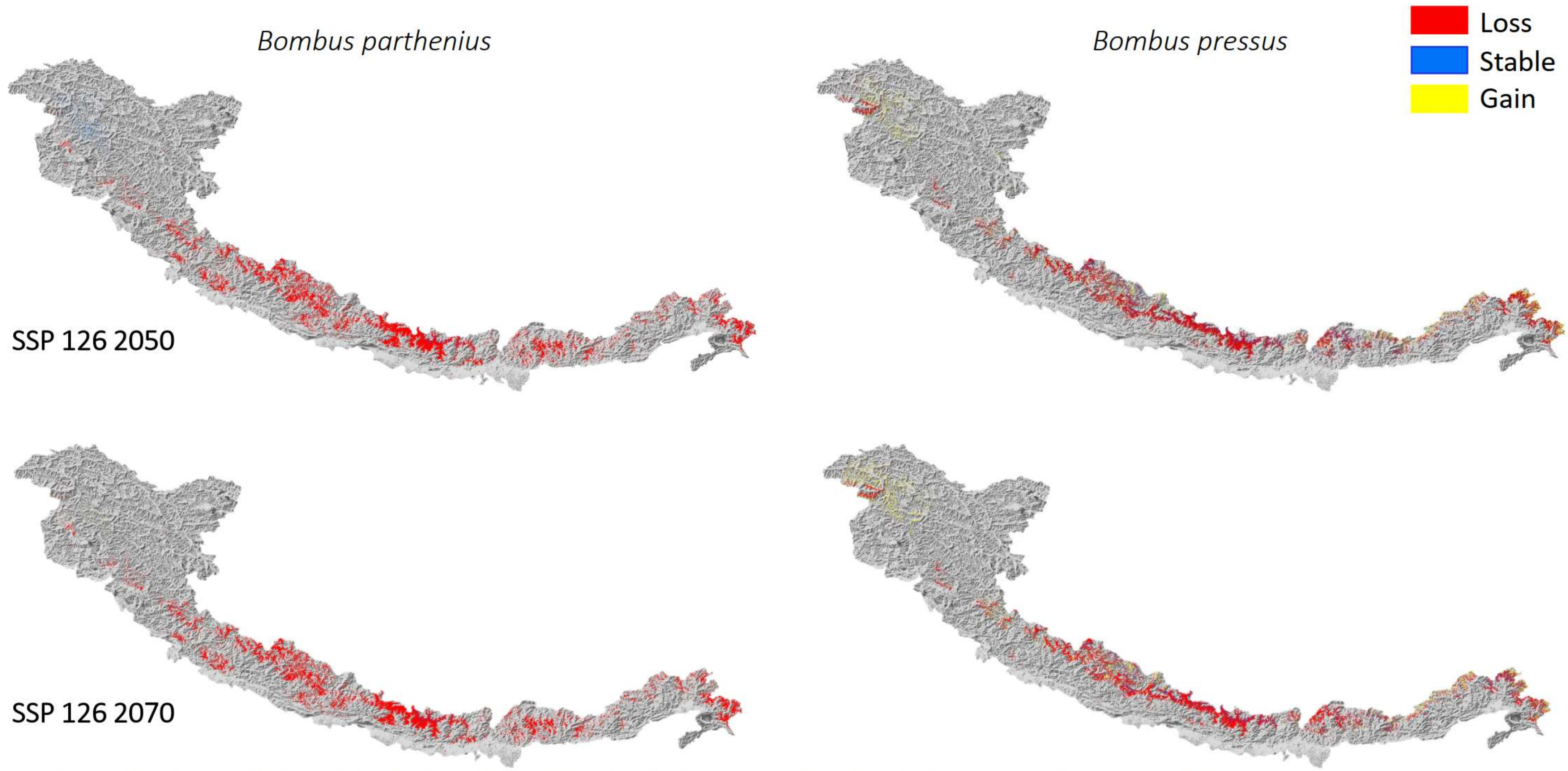

Supplementary figure 14: Predicted future (in 2050 and 2070) habitat suitability of *Bombus parthenius* and *Bombus pressus* in the Himalaya. The figure was generated using open source QGIS software version 3.28.11 (https://www.qgis.org/en/site/forusers/download.html).

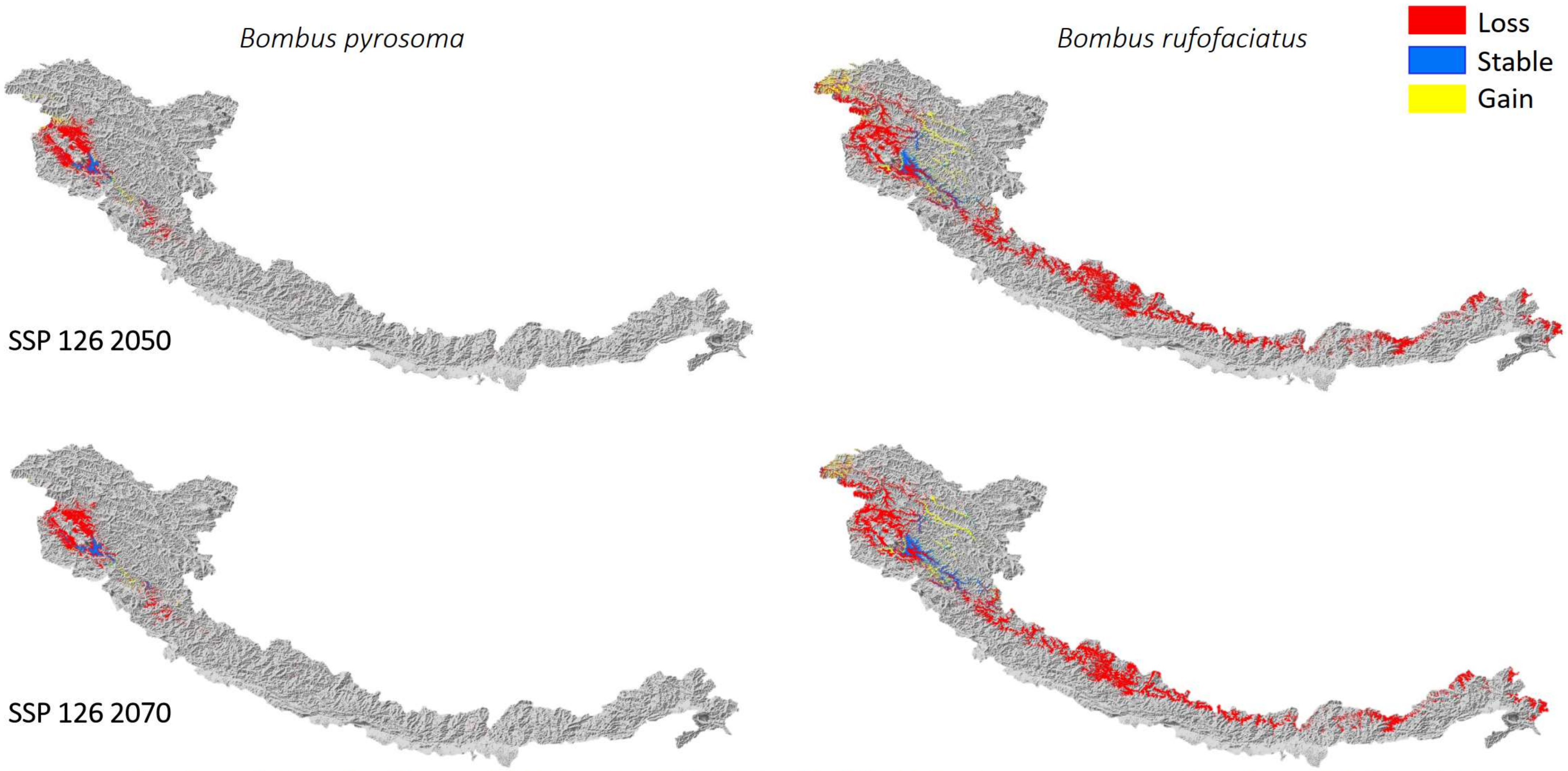

Supplementary figure 15: Predicted future (in 2050 and 2070) habitat suitability of *Bombus pyrosoma* and *Bombus rufofaciatus* in the Himalaya. The figure was generated using open source QGIS software version 3.28.11 (https://www.qgis.org/en/site/forusers/download.html).

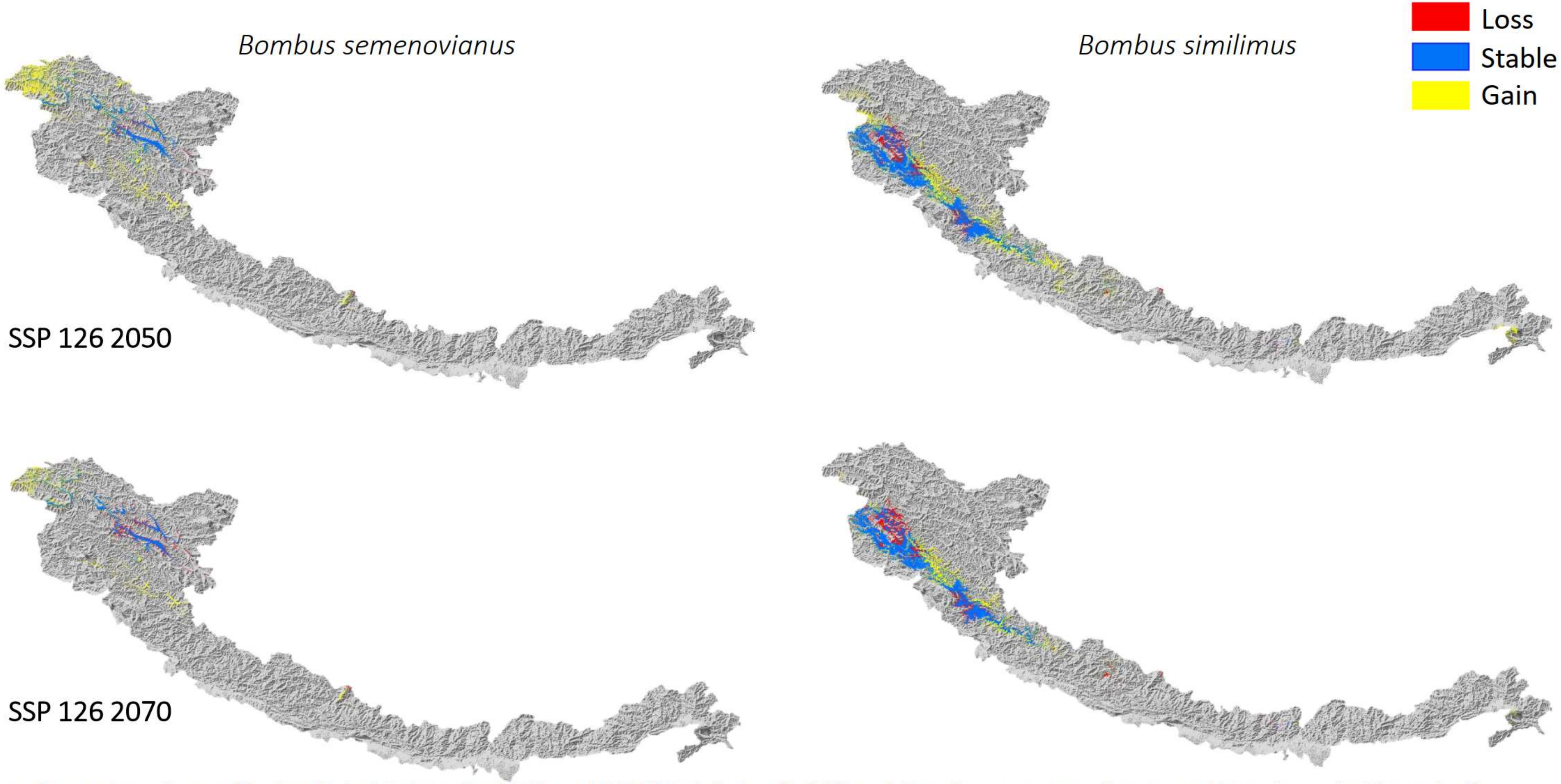

Supplementary figure 16: Predicted future (in 2050 and 2070) habitat suitability of *Bombus semenovianus* and *Bombus similimus* in the Himalaya. The figure was generated using open source QGIS software version 3.28.11 (https://www.qgis.org/en/site/forusers/download.html).

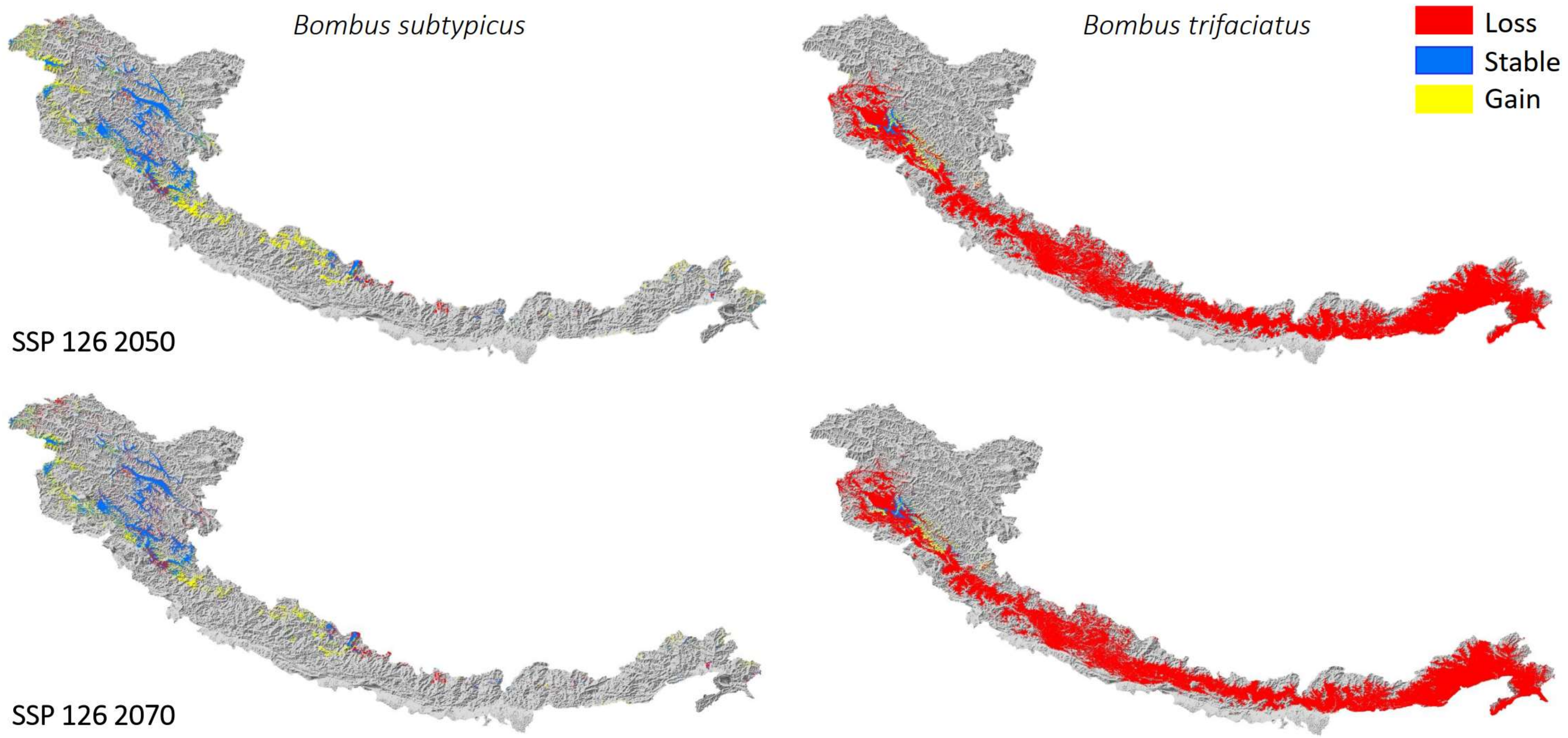

Supplementary figure 17: Predicted future (in 2050 and 2070) habitat suitability of *Bombus subtypicus* and *Bombus trifaciatus* in the Himalaya. The figure was generated using open source QGIS software version 3.28.11 (https://www.qgis.org/en/site/forusers/download.html).

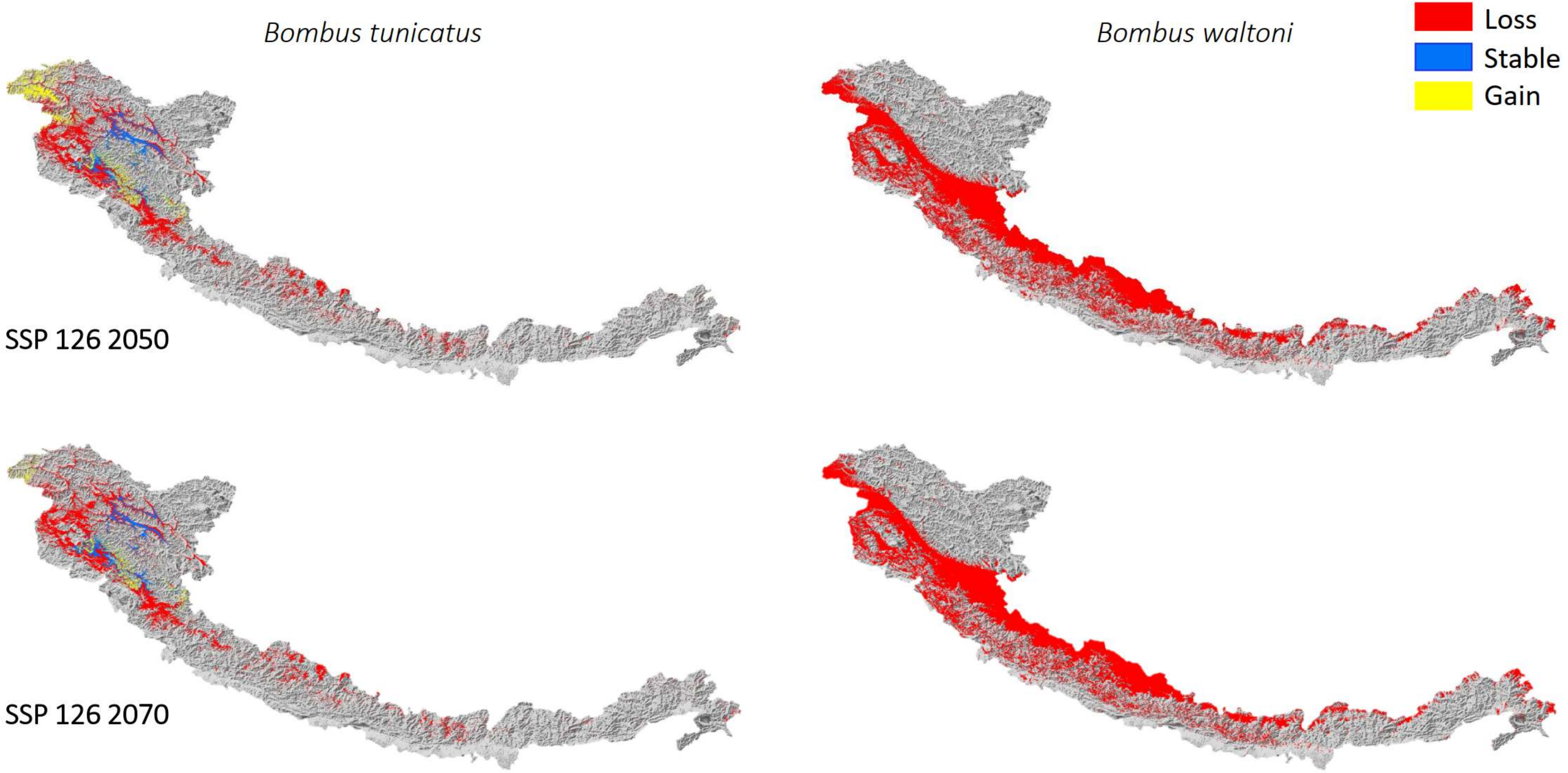

Supplementary figure 18: Predicted future (in 2050 and 2070) habitat suitability of *Bombus tunicatus* and *Bombus waltoni* in the Himalaya. The figure was generated using open source QGIS software version 3.28.11 (https://www.qgis.org/en/site/forusers/download.html).
